# Innate immune cells in chimeric antigen receptor therapy

**DOI:** 10.1016/j.ymthe.2025.10.003

**Published:** 2025-10-06

**Authors:** Marius Jassaud, Lydia Ziane-Chaouche, Marie Duhamel, Michel Salzet

**Affiliations:** 1 Université de Lille, INSERM, CHU Lille, U1192 - Protéomique Réponse Inflammatoire Spectrométrie de Masse (PRISM), Equipe Labellisée Ligue Contre le Cancer, 59000 Lille, France; 2 Institut Universitaire de France, Ministère de l’Enseignement Supérieur, de la Recherche et de l’Innovation, 1 Rue Descartes, 75231 Paris Cedex, France

**Keywords:** innate immune cells, macrophages, natural killer, chimeric antigen receptor, solid cancer

## Abstract

Chimeric antigen receptor (CAR) therapies have revolutionized cancer treatment, particularly with the success of CAR-T cells in hematologic malignancies. However, their application to solid tumors remains limited by major challenges, including cytokine release syndrome, neurotoxicity, poor tumor infiltration, antigen heterogeneity, and high manufacturing costs. These limitations have prompted growing interest in alternative immune effector cells. Innate immune cells, such as natural killer (NK) cells, macrophages, invariant NK T cells, γδ T cells, dendritic cells, and neutrophils, offer distinct advantages. They are associated with a lower risk of graft-versus-host disease, possess intrinsic tumor-homing and cytotoxic properties, and are suitable for off-the-shelf therapeutic platforms. This review explores the biological rationale and clinical potential of CAR-engineered innate immune cells, highlighting key findings from preclinical and clinical studies. Finally, we discuss combinatorial strategies and future directions that could shape the next generation of CAR-based therapies for solid tumors.

## Graphical abstract

**Figure:**
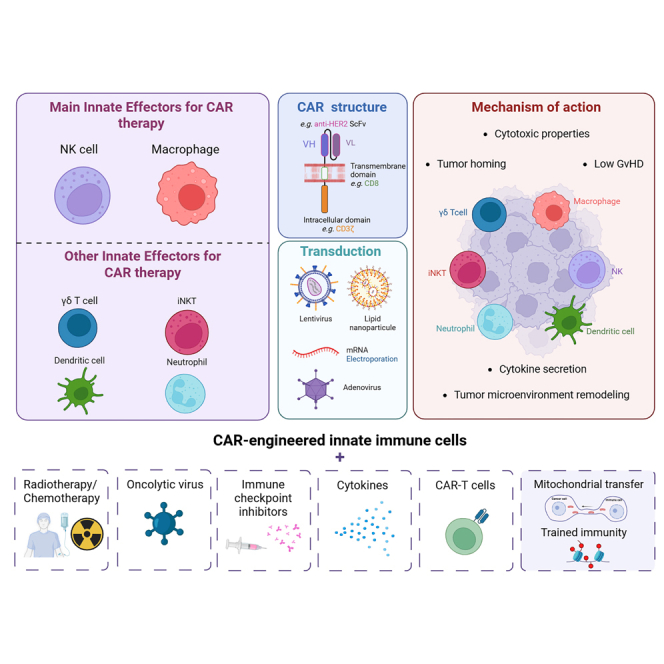

## Teaser

Chimeric antigen receptor (CAR)-engineered innate immune cells represent a promising new frontier for solid tumor treatment, overcoming limitations of CAR-T therapies such as toxicity and poor tumor infiltration. This review highlights their biological advantages and clinical potential, paving the way for innovative, off-the-shelf cancer immunotherapies.

## Introduction

Chimeric antigen receptor (CAR) therapies have emerged as a transformative approach in cancer immunotherapy. Initially conceptualized in the late 1980s, CARs are synthetic receptors designed to redirect immune cells toward tumor-specific antigens in a manner that is independent of major histocompatibility complex (MHC) recognition.^1^^,^^2^^,^^3^^,^^4^ First-generation CARs, composed of an extracellular single-chain variable fragment (scFv) fused to the CD3ζ signaling domain, demonstrated limited clinical efficacy due to suboptimal T cell activation and poor persistence.^4^

To address these shortcomings, second-generation CARs incorporated co-stimulatory domains such as CD28 or 4-1BB, resulting in enhanced proliferation, survival, and cytotoxicity of CAR-T cells.^5^^,^^6^ These innovations led to the remarkable clinical success of CD19-directed CAR-T cell therapies in B cell malignancies, including relapsed acute lymphoblastic leukemia and chemotherapy-refractory chronic lymphocytic leukemia.^7^ The approval of therapies such as tisagenlecleucel^8^ and axicabtagene ciloleucel^9^ marked a major milestone in personalized cancer treatment. Subsequently, third-generation CARs combined multiple co-stimulatory signals,^10^^,^^11^ while fourth-generation CARs—so-called TRUCKs^12^—were engineered to secrete proinflammatory cytokines upon antigen recognition, with the aim of reshaping the tumor microenvironment (TME).

Despite these advances, CAR-T cell therapies face major limitations. Severe side effects, such as cytokine release syndrome (CRS) and neurotoxicity, can compromise safety and require intensive management.^13^ Furthermore, the autologous nature of most CAR-T cell products introduces high manufacturing costs, complex logistics, and delays in treatment initiation, which limit accessibility.

Most critically, extending the success of CAR therapies to solid tumors remains particularly difficult. The solid TME presents numerous barriers, including heterogeneous antigen expression, immunosuppressive signaling, and physical obstacles such as dense extracellular matrix components that impair T cell infiltration and function.^14^ These factors collectively reduce the efficacy of CAR-T cells in solid tumor contexts and underscore the need for alternative strategies.

In response to these challenges, growing interest has emerged around the use of innate immune effector cells, such as natural killer (NK) cells, macrophages, invariant NK T (iNKT) cells, γδ T cells, and dendritic cells (DCs), as alternative platforms for CAR engineering. These cells possess intrinsic tumor-killing capabilities, can operate independently of MHC restriction, and are associated with a lower risk of graft-versus-host disease (GvHD), making them ideal candidates for off-the-shelf allogeneic CAR therapies.

This review explores the rationale and therapeutic potential of CAR-engineered innate immune cells, evaluates the preclinical and clinical data supporting their use, and discusses current challenges. In doing so, we aim to highlight how these alternative cellular platforms may overcome key limitations of CAR T cell therapies—particularly in the treatment of solid tumors.

## Rationale for harnessing innate immune cells

Innate immune cells play a fundamental role as the first line of defense against pathogens and malignant transformations. Unlike adaptive immune cells, which require antigen-specific recognition and clonal expansion, innate immune cells provide rapid and broad-spectrum responses through pattern-recognition receptors (PRRs) and other innate immune mechanisms. Given these attributes, their role in the TME has become an area of significant interest in cancer immunotherapy.

In the context of tumor immunity, various innate immune cell populations, including macrophages, NK cells, DCs, granulocytes, myeloid-derived suppressor cells (MDSCs), innate lymphoid cells, and innate-like T cells, have been recognized for their dynamic influence on tumor progression. These cells exhibit dual functionality, with both tumor-promoting and tumor-suppressing activities, contributing to the heterogeneity of the TME. Harnessing the antitumor potential of innate immune cells while mitigating their immunosuppressive effects has emerged as a promising strategy in immunotherapy.

One major limitation of conventional T cell-based therapies is their reliance on antigen-specific recognition to target malignant cells. Tumors exhibit significant heterogeneity, often leading to immune escape by downregulating tumor-associated antigens (TAAs) or MHC molecules. In contrast, innate immune cells possess antigen-independent cytotoxicity and immunomodulatory functions, naturally infiltrate the TME, and are not constrained by MHC restriction, making them valuable candidates for overcoming tumor resistance mechanisms.

CAR-engineered innate immune cells, such as macrophages, and NK cells, are being explored to enhance tumor targeting and cytotoxicity.^15^^,^^16^^,^^17^ Their MHC independence allows for the development of allogeneic or off-the-shelf cell therapies, reducing manufacturing complexities and costs. Additionally, CAR-engineered innate immune cells have shown lower rates of severe toxicities like CRS and neurotoxicity, further boosting their therapeutic potential and tolerability in patients.

Furthermore, innate immune cells contribute to antitumor immunity through multiple mechanisms. NK cells exert direct cytotoxic effects, while macrophages facilitate tumor clearance through phagocytosis. Additionally, innate cells enhance adaptive immunity by processing and presenting tumor antigens to T cells and through antibody-dependent cell-mediated cytotoxicity (ADCC) and antibody-dependent cell-mediated phagocytosis. By integrating innate immune functions into CAR-based approaches, researchers aim to improve therapeutic efficacy, overcome resistance, and minimize toxicities associated with current immunotherapies.

Given their crucial role in tumor surveillance and immunomodulation, innate immune cells represent a promising avenue for next-generation CAR therapies. Their ability to bridge innate and adaptive immunity provides a compelling rationale for their continued development in cancer immunotherapy. Each type of innate immune cell has unique biological features that can be leveraged to improve CAR therapies, which are discussed in the following sections.

## Innate immune cells as CAR platforms

In this section, we focus in detail on NK cells and macrophages as key players in CAR-based innate immunotherapies (Table 1). These cell types have garnered the most attention and development in the field, owing to their critical roles in the innate immune response and their promising therapeutic potential in cancer immunotherapy. NK cells, with their ability to target and kill tumor cells without prior sensitization, and macrophages, which are pivotal in immunomodulation and TME remodeling, have become central to the development of engineered cell therapies. Their relatively advanced status in clinical and preclinical research makes them the primary focus of this review.

**Table 1:** Comparative overview of CAR-engineered NK cells and macrophages

Key parameters	CAR-NK cells	CAR-macrophages
CAR design	- CD3ζ + 4-1BB (classique) - NK-specific (e.g., NKG2D, 2B4, DAP10, DAP12) - cytokine support (e.g., IL-15)	- CD3ζ, FcRγ, Megf10, MerTK, TLR4, Bai1 - co-stimulatory domains (CD80, CD86, 4-1BBL, OX40L) - dual signaling (CD3ζ + TLR) - CD147 for extracellular matrix remodeling
Transduction	- viral: lentivirus (baboon envelope), vesicular stomatitis virus G (suboptimal), cationic polymers to improve efficiency - non-viral: mRNA-LNP, electroporation	- adenoviral vectors (Ad5f35) - lentiviral with Vpx - non-viral: mRNA-LNP, electroporation
Source	- PB-NK (peripheral blood) - UCB-NK (cord blood) - NK-92 cell line - iPSC-derived NK	- monocyte-derived macrophages (PBMCs) - iPSC-derived macrophages
Mechanisms of action	- CAR-dependent cytotoxicity - CAR-independent killing via NK cell receptors (e.g., NKG2D, CD16) - cytokine secretion (IFN-γ, TNF-α)	- phagocytosis upon CAR engagement - M1 polarization - cytokine release (IL-1β, TNF-α) - TME remodeling - antigen presentation
Persistence	- shorter than CAR-T - can be enhanced via IL-15, feeder cells	- limited due to non-proliferative nature - strategies in development (e.g., CAR-mono)

Other innate immune cells, while promising, are less extensively studied and not as far along in clinical development as NK cells and macrophages. Therefore, we provide a brief overview of the emerging potential of these other innate immune cell types.

## NK cells

NK cells are a subset of innate immune cells that account for approximately 5%–15% of peripheral blood mononuclear cells (PBMCs). These cytotoxic lymphocytes are distinct from T cells due to their expression of CD56 and the absence of CD3 and T cell receptor.^18^ NK cells are referred to as “natural” killers because, unlike T cells, they do not require prior antigen sensitization or human leukocyte antigen (HLA) matching to induce cytotoxic responses. Instead, they recognize and eliminate target cells through a combination of activating and inhibitory signals.

NK cells are primarily involved in the destruction of tumor cells, infected cells, and parasites. In addition to their cytotoxic activity, they secrete a wide range of pro-inflammatory and immunosuppressive cytokines, contributing to the regulation of immune responses. NK cells can be activated in two main ways: first, through signals from stressed or infected cells, and second, through the absence of inhibitory signals. Specifically, the downregulation or loss of MHC class I (MHC-I) molecules on tumor or infected cells triggers NK cell activation, as the inhibitory signals provided by the killer immunoglobulin-like receptors (KIRs) on NK cells are not engaged. This mechanism is particularly important in the context of antitumor immunity, as it allows NK cells to target and kill tumor cells that evade T cell-mediated immune responses by downregulating MHC-I. The presence of NK cells in tumors has been associated with a better prognosis in cancer patients.^19^

**Figure 1:**
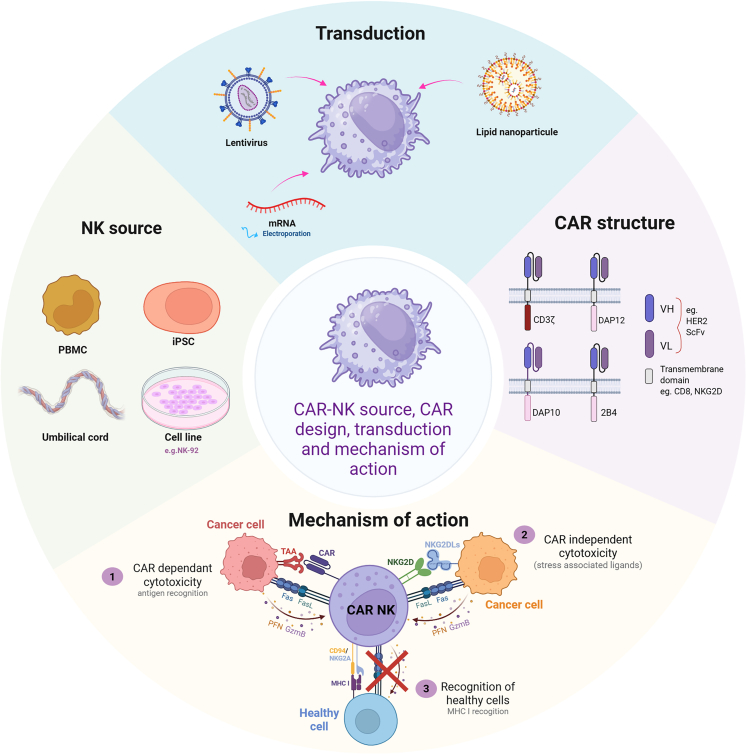
CAR-NK cell generation and mechanisms of action Schematic showing the development of CAR-NKs from different sources, including PBMCs, iPSCs, UCB, and NK cell lines. Delivery of CAR constructs is achieved through viral (e.g., lentivirus) or non-viral (e.g., LNPs, mRNA electroporation) methods. The figure also illustrates CAR-dependent cytotoxicity and CAR-independent killing mediated by natural cytotoxicity receptors.

NK cells exhibit several mechanisms of cytotoxicity. Direct cytotoxicity involves the release of granzymes and perforin, as well as the expression of Fas ligand and tumor necrosis factor (TNF)-related apoptosis-inducing ligand. In addition to direct cytotoxicity, NK cells can contribute to indirect cytotoxicity through the secretion of inflammatory cytokines such as interferon (IFN)-γ, TNF-α, and granulocyte-macrophage-colony-stimulating factor, as well as chemokines like CCL2 and CCL5.^20^ NK cell activation is mediated by a variety of activating receptors, including the natural cytotoxicity receptors NKp30, NKp40, NKp44, and NKp46, which recognize viral, bacterial, and tumor-associated ligands.^21^ NKG2D recognizes ligands that are overexpressed during cellular stress, inflammation, or tumor transformation.^22^ NKG2C recognizes cytomegalovirus peptides presented by the HLA-E molecule.^23^ Additionally, CD16 (FcγRIIIa) is a potent activating receptor that can trigger ADCC independently of other signals.^24^ Due to these diverse activating mechanisms, NK cells are an attractive target for CAR therapy, offering potential advantages over CAR-T cells in overcoming some of their limitations (Figure 1).

### Engineering CAR-NK cells

#### CAR design

The majority of CAR-NK cell studies to date have used second-generation CAR constructs initially developed for CAR-T cells, typically comprising a CD3ζ signaling domain and a 4-1BB co-stimulatory domain. This classical CAR structure has been shown to enhance cytokine production and cytotoxicity in NK cells against tumor targets.^25^ However, given the distinct activation pathways of NK cells compared to T cells, the direct transfer of T cell CAR designs is suboptimal. Consequently, NK cell-specific CAR architectures have been developed to better leverage NK biology.

Several studies have incorporated domains derived from NK-specific activating receptors into CAR design. For instance, Li et al. evaluated 10 CAR variants with CD3ζ signaling domain combined with different transmembrane and co-stimulatory regions from NK receptors, in the context of mesothelin-directed CAR-NK cells. Their optimized construct, incorporating a transmembrane domain from NKG2D and a co-stimulatory domain from 2B4 (CD244), demonstrated superior antitumor activity compared to classical CAR-T cells and CAR-NK designs.^26^ Similarly, another study showed that CAR-NK cells harboring the 2B4 co-stimulatory domain displayed higher anti-CD5 cytotoxicity compared to CAR-NK cells with a 4-1BB co-stimulatory domain.^27^

Other studies have explored the use of intracellular signaling adaptors native to NK cells, such as DAP10 and DAP12. DAP10 has been used in place of traditional co-stimulatory domains like 4-1BB or CD28 to activate NK cells more physiologically.^28^ DAP12, which also contains immunoreceptor tyrosine-based motifs (ITAMs), has even been employed to replace CD3ζ entirely, resulting in increased cytotoxicity in PBMC-derived CAR-NK cells.^29^

Collectively, these innovations underscore that optimizing CAR design for NK cells requires tailoring to their unique signaling machinery. Incorporating NK-specific transmembrane and signaling domains can significantly enhance antitumor responses, potentially surpassing classical CAR constructs adapted from T cell platforms.

#### Transduction

NK cells are notoriously difficult to genetically modify, particularly using lentiviral vectors, which are standard in T cell-based gene therapies. This resistance stems from their innate antiviral defense mechanisms and low expression of key receptors required for viral entry. To overcome these limitations, several strategies have been developed to enhance lentiviral transduction efficiency in NK cells.

One approach involves modifying the cell membrane’s electrostatic properties. The use of cationic polymers such as dextran sulfate have been shown to neutralize the negative charge of the cell membrane, facilitating viral particle entry and improving transduction efficiency.^30^ Another strategy leverages cytokine stimulation and feeder cell co-culture. Supplementing cultures with interleukin-2 (IL-2) not only enhances NK cell expansion but also improves transduction efficiency of primary human NK cells.^31^

The type of viral envelope protein used in lentiviral particles also plays a critical role. While vesicular stomatitis virus G is commonly used, it is suboptimal for NK cells. Colamartino et al. demonstrated that baboon envelope-pseudotyped lentiviral vectors significantly improve gene transfer into NK cells, especially when used with an NK activation and expansion system.^32^

Non-viral strategies have also been explored to introduce the CAR transgene. Golubovskaya et al. developed mRNA lipid nanoparticles (LNPs) capable of efficiently transfecting PBMC-derived NK cells, resulting in CAR-NK cells with strong antitumor activity both *in vitro* and *in vivo*.^33^ Additionally, CAR-NK cells generated by electroporation have been developed and demonstrated effective tumor cell killing.^34^

These findings suggest that tailoring both the gene modification method and the cell activation context is key to successful NK cell genetic modification.

#### Source of cells

In CAR-NK cell therapy, allogeneic NK cells are generally preferred over autologous NK cells. This is because cancer treatments and the immunosuppressive TME can impair the proliferation, cytotoxicity, and functional fitness of patient-derived NK cells.^35^

Several sources have been explored for the generation of allogeneic CAR-NK cells. Peripheral blood human NK cells (PB-NK) can be isolated from healthy donors, but they only make up ∼10% of PBMCs, requiring significant expansion efforts. A higher percentage of NK cells can be isolated from umbilical cord blood (UCB). These cells produce similar levels of cytokines compared to PB-NK cells, although they may show slightly reduced cytotoxic activity.^21^ The continuously growing NK-92 cell line is easy to expand and genetically modify. However, due to genomic instability and potential tumorigenicity, NK-92 cells must be irradiated before use, which limits their *in vivo* persistence to around 48 h.^36^ Induced pluripotent stem cell (iPSC)-derived NK cells offer an expandable, standardized source for CAR-NK production. Studies show that iPSC-derived CAR-NK cells exhibit comparable antitumor activity to PB-derived CAR-NKs in preclinical models, such as ovarian cancer xenografts.^37^

To obtain a sufficient number of functional NK cells, *ex vivo* expansion is essential. The most widely used method involves culturing NK cells with cytokines (e.g., IL-2, IL-15) and irradiated feeder cell lines, including RMI 8866, Epstein-Barr virus-transformed lymphoblastoid cells, and K562 cells engineered to express membrane-bound IL-21 or 4-1BBL.^38^

### Mechanisms of action

Similar to CAR-T cells, CAR-NK cells exert their antitumor effects through CAR-dependent cytotoxicity following the recognition of specific tumor antigens. However, tumor antigen heterogeneity poses a significant challenge; not all tumor cells express the targeted antigen uniformly. This heterogeneity can result in the selective elimination of antigen-positive cells while allowing antigen-negative variants to escape immune surveillance, a phenomenon known as tumor antigen escape.^39^ A distinct advantage of CAR-NK cells lies in their ability to mount CAR-independent cytotoxicity. Unlike CAR-T cells, NK cells possess a broad array of activating receptors that recognize stress-induced ligands often upregulated in tumor cells, including those that have downregulated MHC-I to evade T cell immunity. This natural cytotoxic capacity remains functional in CAR-NK cells and provides an additional mechanism of action that helps circumvent antigen escape.^40^

In addition to their multi-faceted cytotoxic abilities, CAR-NK cells display a superior safety profile compared to CAR-T cells. They produce significantly lower levels of proinflammatory cytokines, such as IL-1 and IL-6, which are major contributors to CRS and neurotoxicity in CAR-T therapy. NK cells primarily secrete lower amounts of IFN-γ, further reducing the risk of severe inflammatory responses.^41^ Moreover, NK cells express inhibitory receptors such as KIRs and NKG2A that bind to self-MHC-I molecules and inhibit NK cell activation. This mechanism underlies their self-tolerance and plays a crucial role in reducing on-target/off-tumor toxicity, a common concern when TAAs are also expressed in normal tissues.^42^ Thus, CAR-NK cells can distinguish malignant from healthy cells more effectively, minimizing collateral damage to normal tissues.^43^

### Clinical trials

Multiple clinical trials on CAR-NK therapy have been launched, with most still ongoing (Table 2). A critical step in translating CAR-NK therapy into the clinic is the development of robust Good Manufacturing Practices (GMP)-compliant manufacturing processes. CAR-NK cells can be produced using automated closed systems such as the CliniMACS Prodigy device, which allows expansion and transduction under GMP conditions while maintaining antitumor activity comparable to small-scale production.^44^ Clinical-grade NK cells can also be expanded using K562 cells engineered to express membrane-bound IL-21, IL-15, and 4-1BBL.^45^^,^^46^ To improve post-cryopreservation viability, pre-treatment with a combination of IL-15 and IL-18 has been shown to rescue NK cells from apoptosis, enhancing their functionality after thawing.^47^

**Table 2:** Clinical trials involving CAR-NK cells in solid tumors

Type of cells	Trial no.	Status	Phase	Disease indication	Antigen
Undisclosed	NCT05137275	recruiting	1	solid tumors, unspecified	5T4
Undisclosed	NCT05194709	recruiting	1	solid tumors, unspecified	5T4
Peripheral blood	NCT05410717	active, recruiting	1	ovarian cancer, testis cancer, endometrial cancer	AXL, CLDN6, GPC3, mesothelin
Cord blood	NCT05703854	recruiting	1/2	renal cell carcinoma, mesothelioma, osteosarcoma	CD70
Cord blood	NCT06464965	recruiting	1	pancreatic cancer, gastric cancer	claudin18.2
Undisclosed	NCT05507593	unknown status	1	small cell lung cancer	DLL3
iPSC	RCT2033200431	recruiting	1	ovarian cancer	GPC3
Undisclosed	NCT06652243	not yet recruiting	undisclosed	hepatocellular carcinoma	GPC3
NK-92	NCT03383978	active, not recruiting	1	Glioblastoma	HER2
Undisclosed	NCT05678205	not yet recruiting	1/2	breast cancer, gastric cancer, gastroesophageal junction adenocarcinoma	HER2
Peripheral blood	NCT03692637	unknown	1	ovarian cancer	mesothelin
Undisclosed	ChiCTR2100048100	not yet recruiting	1	ovarian cancer	mesothelin
iPSC	NCT05395052	terminated	1	non-small cell lung cancer, colorectal cancer, breast cancer, ovarian cancer, pancreatic cancer, head and neck squamous cell carcinoma, gastroesophageal junction adenocarcinoma	MICA/B
NK-92	NCT02839954	recruiting	1/2	hepatocellular carcinoma, non-small cell lung cancer, pancreatic cancer, triple-negative breast cancer, glioblastoma, colorectal cancer, gastric cancer	MUC1
Peripheral blood	NCT03415100	unknown	1	metastatic solid tumors	NKG2DL
Undisclosed	NCT05213195	recruiting	1	colorectal cancer	NKG2DL
NK-92	NCT05528341	recruiting	1	solid tumors, unspecified	NKG2DL
Not yet recruiting	NCT05776355	recruiting	undisclosed	ovarian cancer	NKG2DL
Undisclosed	NCT06478459	not yet recruiting	1	pancreatic cancer	NKG2DL
Undisclosed	NCT06503497	not yet recruiting	1	pancreatic cancer	NKG2DL
NK-92	NCT03656705	completed	1	non-small cell lung cancer	PD-L1
NK-92	NCT03228667	active, not recruiting	2	non-small cell lung cancer, small cell lung cancer, urothelial carcinoma, head and neck squamous cell carcinoma, Merkel cell carcinoma, melanoma, renal cell carcinoma, gastric cancer, cervical cancer, hepatocellular carcinoma, colorectal cancer	PD-L1
NK-92	NCT04050709	active, not recruiting	1	solid tumors, unspecified	PD-L1
NK-92	NCT04390399	active, not recruiting	2	pancreatic cancer	PD-L1
NK-92	NCT04927884	terminated	1/2	triple-negative breast cancer	PD-L1
NK-92	NCT04847466	recruiting	2	gastric cancer, gastroesophageal junction adenocarcinoma, head and neck squamous cell carcinoma	PD-L1
NK-92	NCT06061809	recruiting	2	glioblastoma	PD-L1
NK-92	NCT06161545	not yet recruiting	2	head and neck squamous carcinoma	PD-L1
NK-92	NCT06239220	recruiting	2	head and neck squamous carcinoma	PD-L1
iPSC	NCT03692663	unknown	1	metastatic castration-resistant prostate cancer	PSMA
NK-92	NCT03940820	unknown	1/2	solid tumors, unspecified	ROBO1
NK-92	NCT03941457	unknown	1/2	pancreatic cancer	ROBO1
Cord blood	NCT06066424	recruiting	1	non-small cell lung cancer, breast cancer	TROP2
Cord blood	NCT05922930	recruiting	1/2	pancreatic cancer, ovarian cancer, adenocarcinoma	TROP2
Cord blood	NCT06358430	recruiting	1	colorectal cancer	TROP2
Undisclosed	NCT06454890	not yet recruiting	1/2	non-small cell lung cancer	TROP2
Undisclosed	NCT05686720	unknown	1	triple-negative breast cancer	undisclosed
Undisclosed	NCT05845502	terminated	undisclosed	hepatocellular carcinoma	undisclosed
Undisclosed	NCT05856643	recruiting	1	ovarian cancer	undisclosed
Undisclosed	NCT06572956	active, not recruiting	1	solid tumors, unspecified	undisclosed
Undisclosed	NCT06856278	not yet recruiting	1/2	anaplastic thyroid cancer	undisclosed
Undisclosed	NCT06816823	not yet recruiting	1	pancreatic cancer	undisclosed
Peripheral blood	NCT06773091	recruiting	1	solid tumors, unspecified	undisclosed

For hematopoeitic malignancies, CAR-NK cells target surface markers such as CD19, BCMA, CD33, CD22, and CD7. A notable trial (NCT03056339) using UCB-derived CAR-NK cells targeting CD19 in high-risk B cell malignancies showed promising results: 7 out of 11 patients achieved complete remission. Importantly, this was achieved without major adverse events like CRS, neurotoxicity, or GvHD, demonstrating both efficacy and safety.^16^

In solid tumors, CAR-NK therapies have explored various targets such as ROBO1, MUC1, and 5T4 (NCT03940820, NCT02839954, and NCT05137275, respectively), PSMA for prostate cancer (NCT03692663), and mesothelin for ovarian cancer (NCT03692637). One trial involving intracranial injection of human epidermal growth factor receptor 2 (HER2)-targeted CAR-NK92 cells in recurrent glioblastoma showed no CRS or neurotoxicity, suggesting the approach is safe even in difficult-to-treat brain tumors.^48^ Additionally, CD8^+^ T cell infiltration was observed, indicating an immune-activating effect. However, the treatment extended median progression-free survival by only 7 weeks, underlining the current limitations of CAR-NK in solid tumor applications.

### Limitations

Like CAR-T cells, CAR-NK face several challenges, particularly in solid tumors. One major limitation of CAR-NK therapies is the persistence of infused cells *in vivo*. While the low persistence of CAR-NK cells may help limit side effects, it also reduces the treatment’s efficacy. Tumor trafficking and infiltration are additional limiting factors for CAR-NK therapy and need to be improved to enhance the ability of CAR-NK cells to target and kill tumor cells. The immunosuppressive cytokines and cells within the TME can impair the activity of CAR-NK cells by reducing their cytotoxic activity, limiting their production of pro-inflammatory cytokines, and restricting their expansion and persistence.

## Macrophages

**Figure 2:**
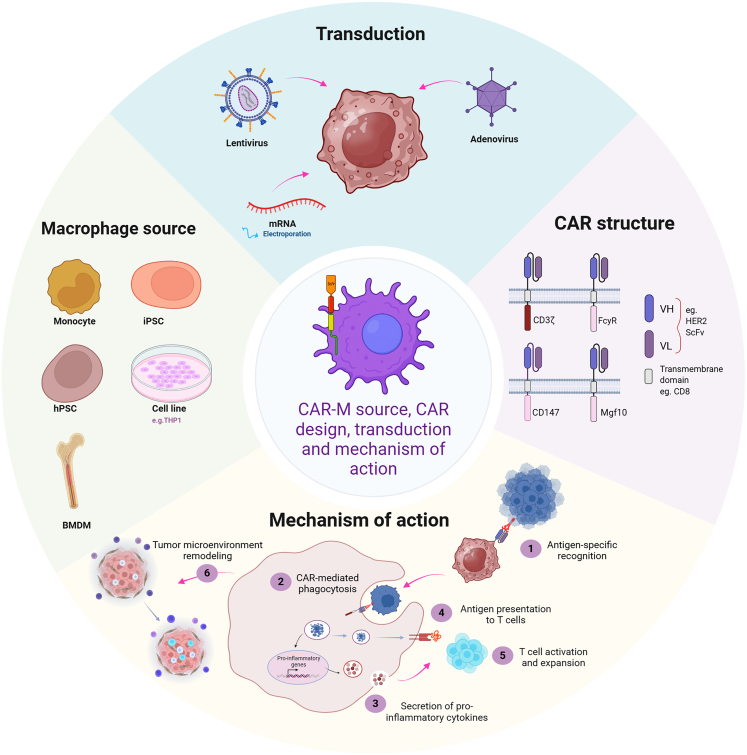
CAR-macrophage generation and mechanisms of action Schematic representation of CAR-M development from various sources such as PBMCs, BMDMs, and hPSCs. Gene transfer methods include viral (lentiviral, adenoviral) and non-viral (mRNA, electroporation) approaches. The modular CAR structure is depicted along with key antitumor functions of CAR-Ms in the tumor microenvironment, including phagocytosis, antigen presentation, cytokine secretion, and T cell activation.

Macrophages are highly versatile innate immune cells that play a critical role in both innate and adaptive immune responses. These cells are capable of phagocytosis, antigen presentation, and the secretion of various cytokines, making them key players in the regulation of immune responses. In the context of cancer, macrophages are found in the TME, where they often adopt an immunosuppressive, pro-tumoral “M2-like” phenotype. This phenotype is associated with promoting tumor growth, metastasis, and an immunosuppressive environment that hampers effective antitumor immunity.^49^ Macrophages can be engineered with CARs to reprogram them into potent antitumor agents. By targeting TAAs and enhancing their pro-inflammatory, tumor-killing abilities, CAR-macrophages (CAR-M) offer a promising therapeutic approach to overcome the challenges of the TME. In addition to their direct tumor-killing capabilities, CAR-M can modulate the immune environment by secreting inflammatory cytokines, such as TNF-α, IL-1β, and IL-6, which can promote the activation of other immune cells and recruit additional effector cells to the tumor site. Furthermore, macrophages are able to engage in immune cell cross-talk, thereby enhancing the overall antitumor immune response. Engineering macrophages with CARs to overcome their pro-tumoral functions and direct them toward tumor destruction represents a promising strategy to improve cancer immunotherapy (Figure 2).

### Engineering CAR-M

#### CAR design

CAR-M represent a novel and promising approach to cancer immunotherapy, particularly for solid tumors where conventional CAR-T therapies often fall short. While the general CAR construct in CAR-M remains similar to that of CAR-T cells, comprising an extracellular antigen-recognition domain, a CD8α hinge and transmembrane domain, and a signaling domain, the intracellular signaling elements can require specific adaptation to macrophage biology. The key divergence from CAR-T design lies in the signaling domain. Unlike T cells, macrophages utilize different intracellular cascades to induce phagocytosis and inflammatory responses. Multiple intracellular domains have been tested to optimize CAR-M function, including FcRγ, Megf10, MerTK, Bai1, and CD3ζ.^50^ These domains share ITAMs that can be phosphorylated by Src family kinases, triggering phagocytic activity. Among these, FcRγ-based CARs have shown the highest levels of phagocytosis, and their efficacy can be enhanced further by tandem domains that recruit phosphatidylinositol 3-kinase. Although CD3ζ is not endogenously expressed in macrophages, CAR-Ms engineered with a CD3ζ intracellular signaling domain have been shown to mediate antigen-specific phagocytosis of tumor cells, highlighting the functional relevance of CD3ζ in driving macrophage effector activity through CAR engagement.^17^^,^^50^

Alternative strategies include using domains from PRRs, such as Toll-like receptor 4 (TLR4). Since TLR4 is naturally expressed on macrophages and responds to pathogen-associated signals, its incorporation into CAR constructs has been shown to boost pro-inflammatory cytokine secretion and resist M2-like polarization within the TME.^51^^,^^52^

Moreover, co-stimulatory domains from the TNF receptor and immunoglobulin superfamily, such as CD80, CD86, 4-1BBL, and OX40L, have been integrated into CAR-Ms to further enhance antigen-dependent phagocytosis and cytokine release.^52^ Dual-signaling CARs, combining CD3ζ with TLR or other innate receptor domains, have been shown to drive M1-like polarization, and enhance tumor phagocytosis, and increase resistance to immunosuppressive signals in the TME.^52^

These design innovations reflect the complexity and potential of CAR-M therapy. By aligning CAR architecture with macrophage-specific biology, researchers can enhance not only direct tumor cell clearance but also broader immune activation and modulation of the TME.

#### Transduction

Macrophages pose a different but equally complex challenge for genetic engineering due to their role as professional phagocytes. These cells actively degrade foreign DNA and viral particles, which hampers traditional transfection methods. Moreover, they express SAMHD1, a deoxynucleoside triphosphate (dNTP) triphosphohydrolase that depletes intracellular dNTPs and inhibits reverse transcription, thereby reducing lentiviral transduction efficiency.^53^

To circumvent SAMHD1-mediated restriction, researchers have employed the accessory protein Vpx, which targets SAMHD1 for proteasomal degradation.^54^ Incorporation of Vpx into lentiviral delivery systems has enabled efficient transduction of primary human macrophages, supporting their use in CAR-M development.^55^^,^^56^

Alternatively, adenoviral vectors offer a non-integrating solution well suited for non-dividing cells like macrophages. In particular, chimeric Ad5f35 vectors, which target the CD46 receptor (abundantly expressed on macrophages), have demonstrated efficient gene delivery and sustained CAR expression.^57^ These vectors also promote a pro-inflammatory, M1-like phenotype, enhancing macrophage antitumor activity.^17^^,^^58^

Non-viral methods have also shown promise. Lipid nanoparticles (LNPs) enable efficient delivery of mRNA encoding CARs into macrophages without the risks associated with genomic integration. These approaches offer transient CAR expression with lower immunogenicity and higher safety profiles. For example, LNP-mediated mRNA transfection has been used to generate both CAR-M and CAR-T cells with potent antitumor activity against B cell lymphoma *in vitro*.^59^ Electroporation of CAR-encoding mRNA is another effective non-viral approach, although its long-term stability in macrophages still requires optimization.^60^

Furthermore, recent studies have demonstrated that *in vivo* generation of CAR-M could be a promising approach. In one study, a cavity-injectable nanoporter-hydrogel, carrying glioma stem cell-targeted CAR genes, was used to generate CAR macrophages/microglia surrounding the cavity to prevent glioblastoma relapse.^61^ A second study employed enucleated mesenchymal stem cells (MSCs) as vehicles for the targeted delivery of CAR-encoding plasmids to reprogram glioma-associated microglia/macrophages. The intrinsic apoptosis of enucleated MSCs triggered macrophage-specific endocytosis, enabling precise delivery of CAR plasmids to macrophages.^62^ A third approach used *in vivo*-injected nanocomplexes consisting of macrophage-targeting nanocarriers and CAR-IFN-γ-encoding plasmid DNA to induce CAR-M1 macrophages.^63^ Additionally, peptide-modified LNPs have been developed for transient, *in situ* programming of macrophages.^64^

Collectively, these diverse strategies, ranging from viral vector engineering to nanoparticle delivery, demonstrate significant progress in overcoming the transduction challenges associated with macrophages and pave the way for scalable and clinically viable CAR-M therapies.

#### Source of cells

CAR-Ms can be generated from various cell types, each with its own advantages and limitations. Immortalized macrophage-like cell lines, such as mouse RAW264.7 and J774A.1 or the human monocytic THP-1 line, are frequently used in CAR-M research due to their ease of culture and genetic manipulation. These cell lines are ideal for early-stage studies and mechanistic evaluations.^17^^,^^50^^,^^65^ However, as immortalized lines, they are not suitable for clinical applications due to safety and regulatory concerns.

More physiologically relevant models use primary macrophages. In murine studies, bone marrow-derived macrophages (BMDMs) are commonly used. These are obtained by harvesting BM cells and differentiating them *in vitro* using macrophage colony-stimulating factor.^66^ In human studies, monocytes isolated from PBMCs are differentiated into macrophages *ex vivo*.^17^ While these primary cells better reflect *in vivo* macrophage biology, they are challenging to isolate and cannot be expanded, which limits their scalability for clinical use.

PSCs, including human embryonic stem cells (hPSCs) and iPSCs, have emerged as a viable alternative. These cells can be expanded indefinitely and differentiated into macrophages at large scale, making them attractive for standardized, allogeneic CAR-M production. Several studies have demonstrated that iPSC-derived CAR-Ms are functional, capable of phagocytosing tumor cells and producing pro-inflammatory cytokines.^67^^,^^68^^,^^69^ Recent studies describe highly efficient monolayer-based systems for generating high-yield macrophages from hPSCs within a limited time frame. These hPSC-CAR-Ms exhibit potent antitumor activity and resist *in vivo* immunosuppression through activation of innate immune responses using IFN-γ and monophosphoryl lipid A.^70^ Another study applied hPSC-derived CAR macrophages to prostate cancer models, incorporating membrane-bound IL-15 for immune activation and a truncated epidermal growth factor receptor (EGFR) as a suicide switch, further enhancing safety and therapeutic control.^71^

A promising emerging strategy involves monocyte-derived CAR precursors (CAR-mono), which are engineered before differentiation. These precursors express CARs stably and differentiate into pro-inflammatory CAR-Ms with potent antitumor activity. *In vivo*, CAR-mono cells have shown durable persistence, tumor homing capabilities, and efficacy against HER2^+^ solid tumors across multiple models.^72^

### Mechanisms of action

Instead of killing tumor cells via direct cytolysis, CAR-Ms are engineered to phagocytose cancer cells upon recognition of tumor-specific antigens (Figure 1). The engineered receptor enhances their ability to engulf target cells and initiates a cascade of downstream immune activation.^17^^,^^50^^,^^73^ Upon engagement with target cells, CAR-Ms adopt a pro-inflammatory M1-like phenotype, characterized by high expression of inflammatory cytokines such as TNF-α, which contributes to tumor cell apoptosis. For example, CAR-Ms derived from iPSCs and engineered with a CD3ζ-TIR signaling domain have shown M1 polarization and robust antitumor activity following co-culture with cancer cells.^52^

Beyond direct cytotoxicity, CAR-Ms also remodel the TME. They secrete matrix metalloproteinases (MMPs), which degrade the extracellular matrix, facilitating immune cell infiltration and disrupting the physical barriers that protect tumors. For instance, CAR-Ms expressing a CD147 intracellular domain have demonstrated high MMP production, which enhances T cell access to the tumor and limits tumor growth.^65^ This M1 phenotype is initiated through antigen recognition and activation of intracellular pathways like MAPK and NF-κB, leading to the secretion of type I IFNs and other cytokines. Furthermore, DNA fragments released from phagocytosed tumor cells activate innate immune pathways such as TLR9 or cGAS-STING, reinforcing M1 polarization and inflammatory signaling.^74^

To enhance their effectiveness, multiple strategies have been employed to promote and stabilize M1 polarization in CAR-M cells. Use of chimeric adenovirus vectors like Ad5f35 has been shown to improve M1 characteristics.^17^ Inhibition of enzymes such as furin, which are involved in immunosuppression, also increases CAR-M cytokine secretion and phagocytic ability, resulting in improved tumor clearance in preclinical models.^58^ Additional approaches involve modifying CARs with signaling domains from innate immune receptors such as TLR4 or IFNGR, which further drive M1 differentiation upon antigen engagement.^51^ Non-genetic methods have also been explored—for example, glycan metabolic labeling combined with click chemistry to create aptamer-functionalized M1 macrophages, which have demonstrated strong antitumor effects in solid tumor models.^75^

### Clinical trials

The clinical translation of CAR-M therapies is still in its early stages, with initial trials concentrating on solid tumors (Table 3). Large-scale GMP-compliant production of CAR-M remains challenging, especially in autologous settings. Monocytes can be isolated and enriched using GMP-grade systems such as the CliniMACS Prodigy, followed by differentiation into macrophages.^76^

**Table 3:** Clinical trials involving CAR-M therapies in solid tumors

Type of cells	Trial no.	Status	Phase	Disease indication	Antigen
Monocytes	NCT06254807	active, not recruiting	1	solid HER2^+^ tumors	HER2
Macrophages	NCT06224738	not yet recruiting	1	gastric cancer	HER2
Macrophages	NCT06562647	recruiting	1	ovarian cancer	mesothelin
Macrophages	NCT04660929	active, not recruiting	1	solid HER2^+^ tumors	HER2

One of the first phase 1 trials (NCT04660929) by Carisma Therapeutics evaluated anti-HER2 CAR-M in patients with HER2^+^ solid tumors.^76^ Results demonstrated a manageable safety profile, with no severe CRS or neurotoxicity. Imaging revealed successful trafficking of CAR-M to tumor sites, accompanied by increased infiltration of endogenous T cells, supporting its potential to modulate the TME. Although efficacy data from this trial remains modest—some patients experienced tumor stabilization as the best response—comprehensive results are still pending. Larger, multi-center trials are needed to validate these findings and determine the broader therapeutic potential of CAR-M in solid tumors.

In 2024, Carisma Therapeutics initiated a second clinical trial (NCT06254807) to evaluate the safety and feasibility of anti-HER2 CAR-mono (CT-0525) in patients with advanced or metastatic HER2-overexpressing solid tumors resistant to standard therapies. This study represents a key step in advancing CAR-mono therapies for solid tumors.

A third clinical trial (NCT06562647) was conducted to evaluate SY001, a CAR-proliferative macrophage-like cell (pMAC) therapy targeting mesothelin, in patients with advanced solid tumors. The study assessed safety, tolerability, pharmacokinetics, and preliminary efficacy. In mesothelin^+^ ovarian cancer, SY001 was administered intravenously in combination with the anti-PD-1 antibody tislelizumab. Patients received multiple doses of SY001, with tislelizumab given prior to the first infusion, supporting the feasibility of combining CAR-pMACs with immune checkpoint blockade.

Another clinical trial (NCT06224738), initiated by the First People’s Hospital of Hangzhou in 2024, aims to evaluate the therapeutic potential of anti-HER2 CAR-M in patients with advanced HER2^+^ gastric cancer, including cases with peritoneal metastasis. In this study, patients’ BM stem cells are mobilized, genetically modified to express a CAR targeting HER2, and subsequently differentiated into CAR-M for intraperitoneal infusion.

### Limits

Like CAR-NK therapies, CAR-Ms face several challenges. Efficient genetic modification remains difficult, although adenoviral vectors have improved transduction. Their limited persistence, due to the non-proliferative nature of macrophages, reduces therapeutic durability. The immunosuppressive TME may reprogram them toward an M2-like phenotype, dampening their antitumor activity. Unlike CAR-T or CAR-NK cells, CAR-Ms lack direct cytotoxicity and instead rely on phagocytosis and immune recruitment, which may be insufficient in poorly immunogenic tumors. Clinical-grade manufacturing also remains complex, and relatively few CAR-M therapies have advanced to clinical trials.

## Other innate immune cells

While NK cells and macrophention for their roles in CAR-based therapies, other innate immune cells also hold considerable promise for enhancing antitumor responses. Among these, iNKT cells, γδ T cells, DCs, and neutrophils are emerging as powerful candidates for CAR engineering (Table 4).

**Table 4:** Clinical trials involving other CAR innate immune cells in liquid and solid tumors

Type of cells	Trial no.	Status	Phase	Disease indication	Antigen
NKT	NCT03294954	recruiting	1	neuroblastoma	GD2
NKT	NCT05487651 NCT03774654	recruiting	1	B cell lymphoma or leukemia	CD19
NKT	NCT06870279 NCT06182735	not yet started	1	clear cell renal cell carcinoma	CD70
NKT	NCT06394622	recruiting	1	CD70^+^ solid tumors	CD70
γδ T cell	NCT05302037	recruiting	1	advanced solid tumors or hematological malignancies	NKG2DL
γδ T cell	NCT04735471	active, not recruiting	1	B cell malignancies	CD20
γδ T cell	NCT06480565	recruiting	1	clear cell renal cell carcinoma	CD70
γδ T cell	NCT06592092	recruiting	1	meningeal metastases	B7H3
γδ T cell	NCT04107142	unknown	1	relapsed or refractory solid tumor	NKG2DL
γδ T cell	NCT04702841	unknown	1	T cell-derived malignancies	CD7
γδ T cell	NCT06150885	recruiting	1	non-small cell lung cancer, triple-negative breast cancer, colorectal cancer, glioblastoma	HLA-G
DC	NCT05631886 NCT05631899	recruiting	1	lymphomas, solid tumors	EphA2
DC	NCT05585996	recruiting	1	B cell lymphoma	CD19

### iNKT cells

iNKT cells are a unique subset of T lymphocytes that share characteristics with both T cells and NK cells. Unlike conventional T cells, which rely on MHC molecules for antigen recognition, iNKT cells recognize glycolipid antigens presented by CD1d, a non-classical antigen-presenting molecule.^77^ Upon activation, iNKT cells rapidly produce large amounts of immunomodulatory cytokines, enhancing DC maturation and αβ T cell differentiation.^78^ They also exert direct cytotoxicity through perforin and granzymes while modulating the TME by eliminating TAMs and MDSCs in a CD1d-dependent manner, thereby counteracting immunosuppression.^79^

The potential of iNKT cells in CAR-based strategies lies in several key advantages. They express a broad range of chemokine receptors, which facilitate their infiltration into tumors and recruitment of other immune effectors.^80^ Furthermore, unlike conventional T cells, iNKT cells do not cause GvHD, making them ideal candidates for off-the-shelf allogeneic therapies.^81^ Engineering iNKT cells with CARs redirects them toward specific tumor antigens, enhancing their therapeutic efficacy. Preclinical studies have demonstrated that CAR-iNKT cells exhibit cytotoxic activity comparable to CAR-T cells, particularly against neuroblastoma and melanoma.^82^^,^^83^ However, their low frequency in PB (0.01%–1% of T cells) and limited persistence *in vivo* pose significant challenges to their clinical application.^84^ To address these limitations, recent work has explored hematopoietic stem/progenitor cells as a renewable source for iNKT generation. A clinically compatible culture system has been established that enables the production of allogeneic CAR-iNKT cells. In addition to CAR expression, these cells can be engineered with immune-enhancing molecules such as IL-15, as well as safety switches and reporter elements, thereby improving their persistence, controllability, and translational potential.^85^

To improve the long-term efficacy of iNKT therapies, several strategies are being explored, including repeated CAR-iNKT cell administrations or cytokine support to enhance their survival and antitumor function.^86^^,^^87^ Additionally, iNKT cells play a pivotal role in reshaping the TME by reducing immunosuppression, particularly through the depletion of M2-like TAMs and activation of DCs, making them attractive candidates for combination therapies. However, a major limitation is that most tumor cells either lack CD1d expression or downregulate it during tumor progression, reducing direct iNKT-mediated recognition.^88^ Optimizing CAR-iNKT strategies, such as enhancing their expansion and persistence through genetic modifications, is therefore crucial for their successful clinical application. Encouraging early clinical data supports the feasibility of CAR-iNKT therapies. A clinical trial (NCT03294954) testing autologous GD2-IL-15 CAR-iNKT cells in pediatric patients with neuroblastoma reported an overall response rate of 25%, with one case of grade 2 CRS.^89^ However, careful monitoring of patients is necessary when using CAR-iNKT cells, as a lethal hyperleukocytosis was observed in this trial, possibly caused by overstimulation of iNKT cells with artificial antigen-presenting cells during manufacturing.^90^

These findings highlight the potential of CAR-iNKT cells as a novel immunotherapeutic approach while underscoring the need for further optimization to enhance their durability and effectiveness in treating solid tumors.

### γδ T cells

γδ T cells represent a distinct subset of T lymphocytes that bridge innate and adaptive immunity.^91^ Unlike conventional αβ T cells, which recognize peptide antigens presented by MHC molecules, γδ T cells recognize a broad range of stress-induced and non-peptidic antigens in an MHC-independent manner. This unique ability allows them to detect and eliminate tumor cells that evade conventional immune responses by downregulating MHC expression. γδ T cells rapidly respond to malignancies through direct cytotoxicity, mediated by perforin and granzymes, as well as through the secretion of pro-inflammatory cytokines such as IFN-γ and TNF-α. Given their potent antitumor properties and their ability to function independently of MHC, γδ T cells are emerging as promising candidates for CAR engineering.

A major advantage of γδ T cells in CAR-based therapies is their broad tumor recognition, enabled by a diverse set of receptors that detect tumor-associated stress proteins.^92^^,^^93^ Unlike αβ T cells, which are restricted by HLA compatibility, γδ T cells function independently of HLA, significantly reducing the risk of GvHD and making them ideal for allogeneic, off-the-shelf applications.^94^ Moreover, γδ T cells naturally reside in epithelial and mucosal tissues, where most solid tumors originate, allowing for efficient tumor infiltration.^95^ The development of CAR-γδ T cells aims to enhance their tumor-targeting capabilities while maintaining their natural advantages. Genetic engineering strategies, such as cytokine support or co-expression of immunostimulatory factors, are being explored to improve their persistence and efficacy. A notable example is the Vδ1-based CAR T cell product, which incorporates a glypican-3 (GPC3)-specific CAR and IL-15 secretion. IL-15 enhances the expansion, survival, and prolonged antitumor activity of CAR-γδ T cells, as demonstrated in preclinical models of hepatocellular carcinoma.^96^ In addition to direct tumor killing, γδ T cells can act as antigen-presenting cells, further stimulating αβ T cell responses and orchestrating a broader antitumor immune attack.

Despite these advantages, several challenges remain in optimizing CAR-γδ T cell therapy. One of the main hurdles is their relatively low abundance in PB, necessitating efficient expansion protocols to generate clinically relevant doses. Additionally, strategies to enhance their persistence and resistance to the immunosuppressive TME are critical for maximizing therapeutic efficacy. Future research efforts aim to refine CAR designs, improve manufacturing scalability, and explore combination therapies to fully harness the potential of γδ T cells in cancer immunotherapy. Given their unique biology, intrinsic safety profile, and strong tumor-targeting capabilities, CAR-γδ T cells represent a compelling alternative to traditional CAR-T therapies, offering a promising avenue for next-generation cancer treatments, particularly for solid tumors.

### DCs

DCs represent a highly specialized and heterogeneous group of professional antigen-presenting cells with the unique ability to initiate and modulate adaptive immune responses. Through their capacity to migrate to lymphoid organs and present antigens via MHC-I and -II molecules, DCs can prime naive T cells and re-activate memory T cells, orchestrating both cytotoxic and helper immune responses. In the TME, DCs are capable of sensing damage-associated molecular patterns (DAMPs), which drive their maturation and functional polarization toward either immunogenic or tolerogenic states. While DCs have been extensively exploited in the context of cancer vaccines, especially via *ex vivo* loading of tumor antigens to boost T cell responses, their therapeutic use has been limited by the immunosuppressive nature of the TME and challenges in DC recruitment, maturation, and persistence.^97^ In an effort to overcome these barriers, an emerging strategy involves equipping DCs with CARs.^98^^,^^99^^,^^100^^,^^101^ This novel approach seeks to combine the highly specific tumor-targeting ability of CARs with the potent T cell-priming functions of DCs, resulting in a cellular product capable not of direct cytotoxicity, but of epitope spreading and durable antitumor immunity. Their ability to produce immunostimulatory cytokines such as IL-12 and to interact with other innate immune cells like NK cells and macrophages further contribute to the amplification of a broad and coordinated immune response. Despite their promise, the clinical translation of CAR-DCs faces significant hurdles. DCs are scarce in PB and exhibit limited proliferative capacity, complicating their large-scale production. Monocyte-derived DCs remain the most commonly used in clinical settings, although their immunogenic potential may not fully recapitulate that of primary DC subsets. The development of universal off-the-shelf CAR-DC products, potentially derived iPSCs, is under exploration but remains in the early stages.^99^ Additionally, the immunosuppressive TME continues to pose a challenge, dampening DC function and reducing their capacity to initiate effective immune responses.

CAR-DCs represent a promising frontier in cellular immunotherapy, especially as a complement to T cell-based strategies. By leveraging their ability to activate and shape adaptive immunity, CAR-DCs offer a path toward more durable and systemic antitumor responses.

### Neutrophils

Neutrophils, the most abundant circulating leukocytes in humans, play a critical role in the immune response to various infections and cancers. Upon entering the TME, neutrophils can become tumor-associated neutrophils, which exhibit significant heterogeneity and plasticity.^102^ While they can exert both pro-tumor and antitumor effects, the predominance of neutrophils in the TME is often associated with immunosuppressive activities that contribute to cancer progression and therapeutic resistance. These cells, particularly those polarized to N2-like phenotypes, can drive angiogenesis, extracellular matrix remodeling, metastasis, and immunosuppression, complicating the effectiveness of cancer therapies. However, neutrophils also possess innate antitumor capabilities, similar to macrophages, and can play a significant role in cancer defense through mechanisms such as phagocytosis, production of reactive oxygen species, and the formation of neutrophil extracellular traps.

In recent years, researchers have turned to CAR engineering as a strategy to enhance the antitumor properties of neutrophils, effectively shifting their phenotype from the N2-like, tumor-promoting state to a more pro-inflammatory, N1-like state.^103^ CAR-engineered neutrophils (CAR-Ns) have shown promising results in preclinical studies, where they demonstrated potent antitumor activity through direct tumor lysis, phagocytosis, and secretion of pro-inflammatory cytokines, while also stimulating other immune cells within the TME. By incorporating CAR constructs, neutrophils are directed to specific tumor antigens, thereby increasing their efficacy in targeting and eliminating cancer cells. This approach harnesses the natural ability of neutrophils to migrate to tumors, potentially making them more efficient than other immune cells in targeting solid tumors.

Despite these advantages, the use of neutrophils in CAR therapies presents several challenges, particularly those related to their limited lifespan and difficulty in expanding *ex vivo*. Neutrophils are terminally differentiated cells, which makes it difficult to obtain sufficient quantities for therapy, particularly from patients. However, innovative strategies have been developed to circumvent this issue. For instance, researchers have genetically engineered hPSCs to produce CAR-N, which demonstrated potent antitumor effects both *in vitro* and *in vivo*.^103^^,^^104^^,^^105^ This approach opens the door for more scalable production of CAR-N and their potential use in clinical settings.

The versatility of neutrophils, combined with their natural tumor-homing ability, makes them an attractive alternative for cellular immunotherapy. However, ensuring that neutrophils retain their antitumor functions and avoiding their potential pro-tumor behaviors through genetic modifications are key for the successful application of CAR-N therapies.

## Combined therapy

**Figure 3:**
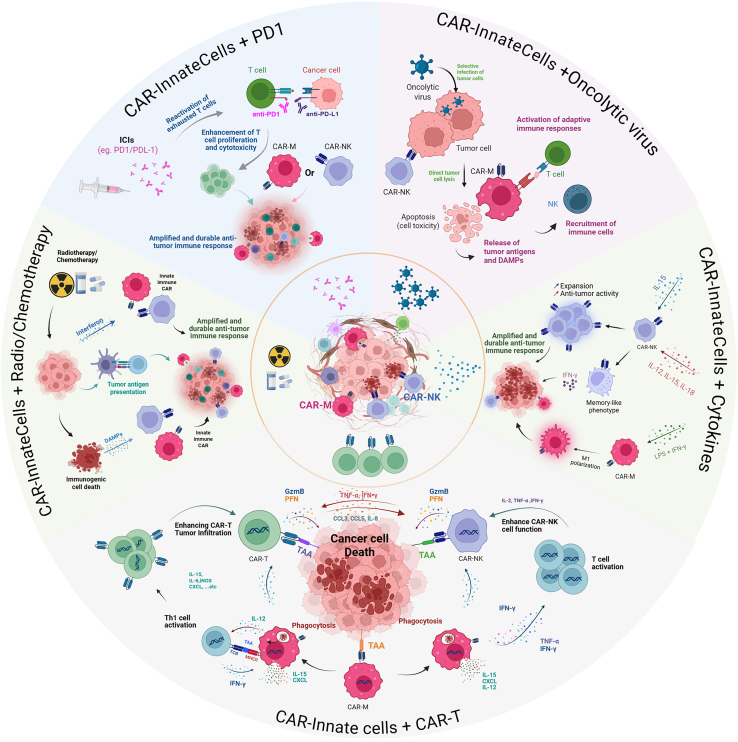
Combination strategies for CAR-innate cell therapies Overview of approaches designed to enhance CAR-engineered innate immune cells, including CAR-Ms and CAR-NKs. The figure includes combination with chemotherapy/radiotherapy, checkpoint inhibitors, oncolytic viruses, cytokine support, and co-administration with CAR-T cells to improve antitumor activity and overcome resistance mechanisms.

Combining CAR-engineered innate immune cells with complementary therapeutic strategies holds great promise for enhancing their efficacy and overcoming current limitations. Integrating these therapies with agents like chemotherapy, cytokines, immune checkpoint inhibitors (ICIs), or oncolytic viruses (OVs) can help modulate the tumor milieu, boost cell function, and improve overall therapeutic outcomes (Figure 3).

### Combining CAR-innate immune cells with conventional cancer therapies

Chemotherapy is typically the first-line treatment for the majority of cancers, but it may have limited efficacy depending on the tumor type or grade. In cancers such as breast and non-small cell lung cancer, neoadjuvant treatments have been shown to consistently increase tumor-infiltrating lymphocytes and CD8^+^ T cells.^106^ Anthracycline-based protocols in breast cancer activate IFN pathways, producing an IFN gene signature that correlates with favorable treatment response.^107^ These chemotherapy-induced effects on the immune microenvironment can synergize with the intrinsic properties of innate immune CAR cells.

For CAR-NK therapy, the combination of CAR-NK cells and cisplatin has demonstrated improvements in the elimination of cancer stem cells, which are more resistant to chemotherapy.^108^ Combining chemotherapy with CAR-M is also promising, as Zheng et al. showed that CAR-M potentiate five different chemotherapies to eradicate pancreatic cancer cell lines.^109^

Radiotherapy works by inducing DNA damage tumors, which not only cause direct cytotoxicity but also trigger immune responses that could complement CAR therapy. Radiotherapy can cause immunogenic cell death, releasing DAMPs like calreticulin, which NK cells can recognize, or ATP, which can be recognized by several immune cell types.^110^ Additionally, radiotherapy enhances tumor antigen presentation by activating DCs.^111^ Studies have shown that high doses of radiation (8 Gy) significantly improved the efficacy of CAR-NK92 cells *in vivo* by increasing the expression of NK cell-activating ligands. While no studies have yet investigated the combination of CAR-M therapy and radiotherapy, the ability of macrophages to recognize stress signals and secrete pro-inflammatory cytokines makes them a promising candidate for this therapeutic approach.

### Combining CAR-innate immune cells with cytokine-based therapies

The use of cytokines can enhance the activity of innate immune cells, particularly NK cells. The combination of IL-15 agonists with CAR-NK cells significantly improves their proliferation and *in vivo* activity.^112^^,^^113^ Pretreating NK cells with a cytokine cocktail (IL-2, IL-15, and IL-18) has been shown to induce a memory-like phenotype. These memory-like CAR-NK cells are more effective than standard CAR-NK cells in NK-resistant B cell lymphoma.^114^^,^^115^ Activity of CAR-M can also be enhanced by the use of cytokine. Huo et al. pre-treated CAR-M with lipopolysaccharides coupled with IFN-γ and showed that this combination deeply polarized CAR-M in the pro-inflammatory state and improved their cytotoxicity.^116^

### Combining CAR-innate immune cells with ICIs

To further advance CAR therapies, targeting immune checkpoints that negatively regulate immune cell functions is a promising strategy. Encouraging results from ICIs in preclinical and clinical settings suggest that combining these inhibitors with CAR-NK cells could enhance tumor regression and improve cancer control compared to monotherapies.

Although programmed cell death protein 1 (PD-1) expression on NK cells is generally low, CAR-NK cells produce substantial levels of IFN-γ, which can induce adaptive immune resistance by upregulating programmed death-ligand 1 (PD-L1) expression on tumor cells. In this context, the use of anti-PD-L1 or anti-PD-1 monoclonal antibodies may counteract this resistance, restore CD8^+^ T cell and NK cell activity, and enhance the antitumor efficacy of CAR-NK cell therapy.^117^ A phase 2 trial of PD-L1-CAR-NK cell therapy combined with the IL-15 agonist N-803 and pembrolizumab (NCT04847466) is under way in the United States to evaluate this combination strategy in patients with recurrent or metastatic solid tumors. Studies have also investigated the combination of CAR-M therapy and PD-1 inhibitors, which significantly enhance tumor growth control and survival while remodeling the TME in preclinical HER2^+^ solid tumor models.^118^ Inhibiting the PD-1/PD-L1 interaction *in vivo* has been shown to enhance macrophage-driven phagocytosis, suppress tumor growth, and extend survival in murine cancer models.^119^

In addition, immune checkpoint receptors like TIGIT, NKG2A, LAG-3, and TIM3 regulate NK cell functions. For instance, third-generation anti-TIM3 CAR-NK cells, developed from iPSCs derived from umbilical cord NK cells, exhibited potent and selective cytotoxicity against TIM3^+^ acute myeloid leukemia cells, sparing TIM3^−^ cells.^120^ This strategy provides a scalable, off-the-shelf immunotherapy targeting for leukemic stem cells, offering improved safety compared to conventional CAR-T therapies. Clinical trials have also evaluated monoclonal antibodies targeting inhibitory KIR2D receptors on NK cells, such as IPH2101. While these agents demonstrated good tolerability and effective receptor saturation, their efficacy as monotherapies was limited.^121^ These results suggest that combining anti-KIR2D antibodies with CAR-NK cell therapies may yield greater therapeutic benefits.

Macrophage-specific immune checkpoints have also been explored. For example, researchers engineered CAR-Ms by fusing a humanized scFv with FcγRIIa and incorporating short hairpin RNA to silence signal regulatory protein α (SIRPα), thereby disrupting the CD47-SIRPα “don’t eat me” signaling pathway. These CAR-short hairpin SIRPα-M cells exhibited an M1-like phenotype, enhanced phagocytosis, potent cytotoxicity against HER2^+^ tumor cells, and the capacity to eliminate patient-derived organoids. *In vivo*, they improved tumor control, extended survival, and promoted T cell infiltration.^122^ Additionally, CAR-Ms engineered with a CD47-blocking component were able to overcome tumor antigen heterogeneity, thereby enhancing phagocytosis and antigen cross-presentation, which further activated T cells and strengthened the antitumor immune response.^123^

### Combining CAR-innate immune cells with OVs

Integrating CAR-NK cell therapy with oncolytic virotherapy offers the potential to synergize the cytotoxic activity of CAR-NK cells with the tumor-selective lysis and immunomodulatory effects of OVs). These viruses can enhance the immunogenicity of the TME, promoting better trafficking, activation, and persistence of CAR-NK cells.^124^ For instance, an HSV-1-based OV expressing a human IL-15/IL-15Rα fusion protein (OV-IL15C) and off-the-shelf EGFR-targeted CAR-NK cells has been shown to enhance glioblastoma multiforme cytotoxicity and improve NK and CD8^+^ T cell survival, promoting NK and CD8^+^ T cell infiltration and CAR-NK persistence *in vivo*.^124^

Similarly, combining CAR-M with OVs offers a promising approach. CAR-M cells can directly phagocytose tumor cells and modulate the TME, while OVs selectively infect and lyse tumor cells,^125^ releasing tumor antigens and pro-inflammatory signals that further stimulate immune responses. Although studies on CAR-M/OV combinations are limited, preclinical data using CAR-T cells and OVs have demonstrated improved tumor control and immune activation, notably in pediatric solid tumor models.^126^ OVs can also be engineered to express immunostimulatory molecules, further enhancing the recruitment and activation of myeloid cells within the TME, which could improve CAR-M functionality. For instance, OVs can be designed to deliver cytokines, chemokines, or co-stimulatory molecules like CD40 ligand and 4-1BB ligand, which have been shown to stimulate DC activation and enhance antitumor immune responses.^127^

Additionally, OVs can be modified to express TAAs, serving as therapeutic vaccines that target and amplify immune responses against these antigens. OVs have also been shown to upregulate MHC and co-stimulatory molecules on tumor cells, increasing their visibility to immune effector cells.^128^

### Combining multiple CAR-engineered immune cells

To improve the efficacy of CAR-based therapies, combining different CAR-engineered immune cells within a single therapeutic strategy is gaining attention. This approach leverages the unique strengths of various immune cell types, improving treatment outcomes and mitigating risks such as CRS and neurotoxicity. CAR-NK cells provide rapid antitumor activity but face limitations in long-term persistence. Conversely, CAR-T cells exhibit sustained antitumor activity but may induce severe toxicities like CRS due to their explosive expansion.

A promising strategy developed by Nkarta Therapeutics combines CAR-NK and CAR-T cells, leading to a more potent and cumulative antitumor response while reducing the cytokine release associated with CAR-T cell therapy and promoting CAR-NK cell expansion. They showed that the presence of CAR-NK cells reduced the proliferation of CAR-T cells, avoiding potential overactivation.^129^

Additionally, CAR-M cells, with their superior ability to infiltrate solid tumors and enhance T cell responses, can complement CAR-T therapy by amplifying antitumor immunity. The interaction between CAR-T and CAR-M cells can enhance their effectiveness, as inflammatory signals from CAR-T cells promote the polarization of CAR-M cells to the M1 phenotype, boosting cytotoxic activity. CAR-T cells also upregulate co-stimulatory ligands on CAR-M cells, which further boosts the activation and effectiveness of CAR-T cells.^66^

Combining multiple CAR-engineered immune cells, such as CAR-NK cells, CAR-T cells, and CAR-M cells, offers an exciting multi-pronged approach that maximizes rapid antitumor activity, sustained immune persistence, and enhanced tumor infiltration. However, several challenges remain before these combination strategies can be fully translated into clinical practice. These challenges include optimizing the scalability and manufacturing processes of multi-cell therapies, ensuring that each engineered immune cell type functions effectively without interfering with the others, and conducting thorough long-term safety evaluations. In addition, the economic burden associated with the development, production, and administration of complex multi-cellular therapies presents a significant barrier to widespread clinical adoption.

## Conclusion and perspectives

CAR-engineered innate immune cells represent a promising frontier in cancer immunotherapy. They offer unique advantages over traditional CAR-T therapies, including off-the-shelf availability, reduced toxicity, enhanced trafficking to tumor sites, and potent antitumor functions. These features make them particularly well suited for addressing one of the greatest limitations of current CAR therapies: the treatment of solid tumors.

This review has outlined the significant progress made in preclinical and early clinical studies, as well as the synergistic potential of combining CAR-innate cells with conventional therapies, cytokines, ICIs, and OVs. These combinatorial approaches may overcome the barriers of tumor heterogeneity, immune evasion, and resistance.

Despite these advances, several challenges remain, including limited persistence, immunosuppressive TMEs, manufacturing scalability, and economic feasibility. Continued innovation in gene editing, cell expansion technologies, and combinatorial strategies will be key to overcoming these obstacles. Another critical limitation, shared by all CAR-based strategies, is the identification of suitable tumor-associated antigens that are both highly specific to cancer cells and broadly expressed across patient populations. The lack of ideal antigens continues to pose a major risk for off-target effects and limits the universality of current CAR approaches.

In parallel, several novel avenues are emerging to further improve CAR-innate immune cell therapies. For example, CAR-mono can be engineered prior to differentiation and may expect robust antitumor activity, enhanced persistence, and superior tumor infiltration. Similarly, iPSC-derived NK cells and macrophages offer a scalable and standardized platform for off-the-shelf CAR products. These sources address key challenges related to consistency and manufacturing logistics. Furthermore, combining CAR-innate cells with complementary treatments, including cytokine support, checkpoint blockade, or CAR-T cell co-administration, may generate synergistic immune responses and help circumvent resistance in solid tumors.

In addition to these strategies, recent advances in immunology have introduced novel biological concepts that could further enhance CAR-innate immune cell functionality. Notably, trained immunity, a memory-like property of innate immune cells, may enable the pre-conditioning of immune cell precursors to induce epigenetic and metabolic reprogramming, thereby boosting their responsiveness and persistence.^130^ Similarly, mitochondrial dysfunction can impair the effector function of immune cells, a process that may be exacerbated by tumor-derived nanotubes that extract mitochondria from immune cells. Mitochondrial transfer from supportive cells has been shown to restore the metabolic fitness and cytotoxic potential of exhausted immune cells.^131^ Applying this approach to CAR-NK or CAR-M could enhance their performance in immunosuppressive tumor environments. These emerging approaches offer a new layer of functional engineering that may synergize with existing CAR platforms.

In parallel, emerging technologies hold great promise for guiding the rational design and application of CAR-innate immune cells. Single-cell technologies and other multi-omics approaches can provide high-resolution insights into tumor heterogeneity, immune cell states, and the TME, thereby enabling the identification of predictive biomarkers for treatment efficacy and resistance. Such tools may also inform the selection of optimal targets and guide patient stratification to maximize therapeutic benefit. Beyond molecular profiling, the choice of immune cell type itself may need to be tailored to tumor context. For example, CAR-NK cells, with their strong cytotoxicity and reduced risk of GvHD, may be particularly suited for hematological malignancies and metastatic tumors, whereas CAR-M, with their capacity to remodel the TME, may be more advantageous in solid tumors characterized by dense stroma and immunosuppression. Similarly, CAR-iNKT cells may be uniquely positioned to bridge innate and adaptive responses, making them attractive for combination regimens.

Continued innovation in synthetic biology, gene editing, and combination strategies will be essential to address these issues. Moreover, advancing our understanding of innate-adaptive immune crosstalk, optimizing cell trafficking and tumor infiltration, and developing predictive biomarkers for patient selection will be key to improving therapeutic outcomes. As these strategies mature, CAR-innate immune cell therapies are poised to become an integral part of the next generation of precision cancer immunotherapy.

## Acknowledgments

This research was supported by grants from the Ministère de l’Enseignement Supérieur et de la Recherche, INSERM, and the Institut Universitaire de France (to M.S.). The authors thank La Ligue Contre le Cancer (PB/IP/IQ-18374), the French National Cancer Institute (INCa-PLBIO21-195), and Agence Nationale de la Recherche (ANR-24-CE18-7403-01) for their support.

## Author contributions

Conceptualization, M.D. and M.S.; investigation, M.J., L.Z.-C., M.D., and M.S.; writing – original draft, M.J., L.Z.C., M.D., and M.S.; writing – review & editing, M.D. and M.S.; figure creation, M.J. and L.Z.-C.; funding acquisition, M.D. and M.S.

## Declaration of interests

The authors declare no competing interests.

## References

[1] Krause A., Guo H.F., Latouche J.B., Tan C., Cheung N.K., Sadelain M. (1998). Antigen-dependent CD28 Signaling Selectively Enhances Survival and Proliferation in Genetically Modified Activated Human Primary T Lymphocytes. J. Exp. Med..

[2] Maher J., Brentjens R.J., Gunset G., Rivière I., Sadelain M. (2002). Human T-lymphocyte cytotoxicity and proliferation directed by a single chimeric TCRζ/CD28 receptor. Nat. Biotechnol..

[3] Pinthus J.H., Waks T., Kaufman-Francis K., Schindler D.G., Harmelin A., Kanety H., Ramon J., Eshhar Z. (2003). Immuno-gene therapy of established prostate tumors using chimeric receptor-redirected human lymphocytes. Cancer Res..

[4] Gross G., Waks T., Eshhar Z. (1989). Expression of immunoglobulin-T-cell receptor chimeric molecules as functional receptors with antibody-type specificity. Proc. Natl. Acad. Sci. USA.

[5] Finney H.M., Lawson A.D., Bebbington C.R., Weir A.N., Weir C. (1998). Chimeric Receptors Providing Both Primary and Costimulatory Signaling in T Cells from a Single Gene Product. J. Immunol..

[6] Brentjens R.J., Latouche J.B., Santos E., Marti F., Gong M.C., Lyddane C., King P.D., Larson S., Weiss M., Rivière I., Sadelain M. (2003). Eradication of systemic B-cell tumors by genetically targeted human T lymphocytes co-stimulated by CD80 and interleukin-15. Nat. Med..

[7] Brentjens R.J., Rivière I., Park J.H., Davila M.L., Wang X., Stefanski J., Taylor C., Yeh R., Bartido S., Borquez-Ojeda O. (2011). Safety and persistence of adoptively transferred autologous CD19-targeted T cells in patients with relapsed or chemotherapy refractory B-cell leukemias. Blood.

[8] Maude S.L., Laetsch T.W., Buechner J., Rives S., Boyer M., Bittencourt H., Bader P., Verneris M.R., Stefanski H.E., Myers G.D. (2018). Tisagenlecleucel in Children and Young Adults with B-Cell Lymphoblastic Leukemia. N. Engl. J. Med..

[9] Locke F.L., Ghobadi A., Jacobson C.A., Miklos D.B., Lekakis L.J., Oluwole O.O., Lin Y., Braunschweig I., Hill B.T., Timmerman J.M. (2019). Long-term safety and activity of axicabtagene ciloleucel in refractory large B-cell lymphoma (ZUMA-1): a single-arm, multicentre, phase 1–2 trial. Lancet Oncol..

[10] Derigs P., Schubert M.L., Dreger P., Schmitt A., Yousefian S., Haas S., Röthemeier C., Neuber B., Hückelhoven-Krauss A., Brüggemann M. (2024). Third-generation anti-CD19 CAR T cells for relapsed/refractory chronic lymphocytic leukemia: a phase 1/2 study. Leukemia.

[11] Li G., Boucher J.C., Kotani H., Park K., Zhang Y., Shrestha B., Wang X., Guan L., Beatty N., Abate-Daga D., Davila M.L. (2018). 4-1BB enhancement of CAR T function requires NF-κB and TRAFs. JCI insight.

[12] Chmielewski M., Abken H. (2015). TRUCKs: The fourth generation of CARs. Expert Opin. Biol. Ther..

[13] Neelapu S.S., Tummala S., Kebriaei P., Wierda W., Gutierrez C., Locke F.L., Komanduri K.V., Lin Y., Jain N., Daver N. (2018). Chimeric antigen receptor T-cell therapy-assessment and management of toxicities. Nat. Rev. Clin. Oncol..

[14] Kankeu Fonkoua L.A., Sirpilla O., Sakemura R., Siegler E.L., Kenderian S.S. (2022). CAR T cell therapy and the tumor microenvironment: Current challenges and opportunities. Mol. Ther. Oncolytics.

[15] Pan K., Farrukh H., Chittepu V.C.S.R., Xu H., Pan C.X., Zhu Z. (2022). CAR race to cancer immunotherapy: from CAR T, CAR NK to CAR macrophage therapy. J. Exp. Clin. Cancer Res..

[16] Liu E., Marin D., Banerjee P., Macapinlac H.A., Thompson P., Basar R., Nassif Kerbauy L., Overman B., Thall P., Kaplan M. (2020). Use of CAR-Transduced Natural Killer Cells in CD19-Positive Lymphoid Tumors. N. Engl. J. Med..

[17] Klichinsky M., Ruella M., Shestova O., Lu X.M., Best A., Zeeman M., Schmierer M., Gabrusiewicz K., Anderson N.R., Petty N.E. (2020). Human chimeric antigen receptor macrophages for cancer immunotherapy. Nat. Biotechnol..

[18] De Maria A., Bozzano F., Cantoni C., Moretta L. (2011). Revisiting human natural killer cell subset function revealed cytolytic CD56dimCD16+ NK cells as rapid producers of abundant IFN-γ on activation. Proc. Natl. Acad. Sci. USA.

[19] Cursons J., Souza-Fonseca-Guimaraes F., Foroutan M., Anderson A., Hollande F., Hediyeh-Zadeh S., Behren A., Huntington N.D., Davis M.J. (2019). A gene signature predicting natural killer cell infiltration and improved survival in melanoma patients. Cancer Immunol. Res..

[20] Guillerey C. (2020). NK Cells in the Tumor Microenvironment. Adv. Exp. Med. Biol..

[21] Myers J.A., Miller J.S. (2021). Exploring the NK cell platform for cancer immunotherapy. Nat. Rev. Clin. Oncol..

[22] Zingoni A., Molfetta R., Fionda C., Soriani A., Paolini R., Cippitelli M., Cerboni C., Santoni A. (2018). NKG2D and Its Ligands: “One for All, All for One.”. Front. Immunol..

[23] Siemaszko J., Marzec-Przyszlak A., Bogunia-Kubik K. (2023). Activating NKG2C Receptor: Functional Characteristics and Current Strategies in Clinical Applications. Arch. Immunol. Ther. Exp..

[24] Bryceson Y.T., March M.E., Ljunggren H.G., Long E.O. (2006). Activation, coactivation, and costimulation of resting human natural killer cells. Immunol. Rev..

[25] Kruschinski A., Moosmann A., Poschke I., Norell H., Chmielewski M., Seliger B., Kiessling R., Blankenstein T., Abken H., Charo J. (2008). Engineering antigen-specific primary human NK cells against HER-2 positive carcinomas. Proc. Natl. Acad. Sci. USA.

[26] Li Y., Hermanson D.L., Moriarity B.S., Kaufman D.S. (2018). Human iPSC-Derived Natural Killer Cells Engineered with Chimeric Antigen Receptors Enhance Anti-tumor Activity. Cell Stem Cell.

[27] Xu Y., Liu Q., Zhong M., Wang Z., Chen Z., Zhang Y., Xing H., Tian Z., Tang K., Liao X. (2019). 2B4 costimulatory domain enhancing cytotoxic ability of anti-CD5 chimeric antigen receptor engineered natural killer cells against T cell malignancies. J. Hematol. Oncol..

[28] Chang Y.H., Connolly J., Shimasaki N., Mimura K., Kono K., Campana D. (2013). A chimeric receptor with NKG2D specificity enhances natural killer cell activation and killing of tumor cells. Cancer Res..

[29] Töpfer K., Cartellieri M., Michen S., Wiedemuth R., Müller N., Lindemann D., Bachmann M., Füssel M., Schackert G., Temme A. (2015). DAP12-Based Activating Chimeric Antigen Receptor for NK Cell Tumor Immunotherapy. J. Immunol..

[30] Nanbakhsh A., Best B., Riese M., Rao S., Wang L., Medin J., Thakar M.S., Malarkannan S. (2018). Dextran enhances the lentiviral transduction efficiency of murine and human primary NK cells. J. Vis. Exp..

[31] Allan D.S.J., Chakraborty M., Waller G.C., Hochman M.J., Poolcharoen A., Reger R.N., Childs R.W. (2021). Systematic improvements in lentiviral transduction of primary human natural killer cells undergoing ex vivo expansion. Mol. Ther. Methods Clin. Dev..

[32] Colamartino A.B.L., Lemieux W., Bifsha P., Nicoletti S., Chakravarti N., Sanz J., Roméro H., Selleri S., Béland K., Guiot M. (2019). Efficient and Robust NK-Cell Transduction With Baboon Envelope Pseudotyped Lentivector. Front. Immunol..

[33] Golubovskaya V., Sienkiewicz J., Sun J., Zhang S., Huang Y., Zhou H., Harto H., Xu S., Berahovich R., Wu L. (2023). CAR-NK Cells Generated with mRNA-LNPs Kill Tumor Target Cells In Vitro and In Vivo. Int. J. Mol. Sci..

[34] Xiao L., Cen D., Gan H., Sun Y., Huang N., Xiong H., Jin Q., Su L., Liu X., Wang K. (2019). Adoptive Transfer of NKG2D CAR mRNA-Engineered Natural Killer Cells in Colorectal Cancer Patients. Mol. Ther..

[35] Tai L.H., Zhang J., Auer R.C. (2013). Preventing surgery-induced NK cell dysfunction and cancer metastases with influenza vaccination. Oncoimmunology.

[36] Suck G., Odendahl M., Nowakowska P., Seidl C., Wels W.S., Klingemann H.G., Tonn T. (2016). NK-92: an ‘off-the-shelf therapeutic’ for adoptive natural killer cell-based cancer immunotherapy. Cancer Immunol. Immunother..

[37] Hermanson D.L., Bendzick L., Pribyl L., McCullar V., Vogel R.I., Miller J.S., Geller M.A., Kaufman D.S. (2016). Induced pluripotent stem cell-derived natural killer cells for treatment of ovarian cancer. Stem Cells.

[38] Baek H.J., Kim J.S., Yoon M., Lee J.J., Shin M.G., Ryang D.W., Kook H., Kim S.K., Cho D. (2013). Ex vivo expansion of natural killer cells using cryopreserved irradiated feeder cells. Anticancer Res..

[39] Majzner R.G., Mackall C.L. (2018). Tumor antigen escape from car t-cell therapy. Cancer Discov..

[40] Oei V.Y.S., Siernicka M., Graczyk-Jarzynka A., Hoel H.J., Yang W., Palacios D., Almåsbak H., Bajor M., Clement D., Brandt L. (2018). Intrinsic functional potential of NK-Cell subsets constrains retargeting driven by chimeric antigen receptors. Cancer Immunol. Res..

[41] Hunter B.D., Jacobson C.A. (2019). CAR T-Cell Associated Neurotoxicity: Mechanisms, Clinicopathologic Correlates, and Future Directions. J. Natl. Cancer Inst..

[42] Lanier L.L. (2008). Up on the tightrope: Natural killer cell activation and inhibition. Nat. Immunol..

[43] Portillo A.L., Hogg R., Poznanski S.M., Rojas E.A., Cashell N.J., Hammill J.A., Chew M.V., Shenouda M.M., Ritchie T.M., Cao Q.T. (2021). Expanded human NK cells armed with CAR uncouple potent anti-tumor activity from off-tumor toxicity against solid tumors. iScience.

[44] Albinger N., Müller S., Kostyra J., Kuska J., Mertlitz S., Penack O., Zhang C., Möker N., Ullrich E. (2024). Manufacturing of primary CAR-NK cells in an automated system for the treatment of acute myeloid leukemia. Bone Marrow Transpl..

[45] Zhang C., Kadu S., Xiao Y., Johnson O., Kelly A., O’Connor R.S., Lai M., Kong H., Srivatsa S., Tai V. (2023). Sequential Exposure to IL21 and IL15 During Human Natural Killer Cell Expansion Optimizes Yield and Function. Cancer Immunol. Res..

[46] Gong S., Mei N., Wang J., Zhu J., Wang L., Lu X., He P., Chen W., Xi L., Bao Y. (2025). A novel feeder cell based on 4-1BBL and membrane-bound IL-21/IL-15 induce highly expansion and anti-tumor effect of natural killer cells. BMC Biotechnol..

[47] Berjis A., Muthumani D., Aguilar O.A., Pomp O., Johnson O., Finck A.V., Engel N.W., Chen L., Plachta N., Scholler J. (2024). Pretreatment with IL-15 and IL-18 rescues natural killer cells from granzyme B-mediated apoptosis after cryopreservation. Nat. Commun..

[48] Burger M.C., Forster M.T., Romanski A., Straßheimer F., Macas J., Zeiner P.S., Steidl E., Herkt S., Weber K.J., Schupp J. (2023). Intracranial injection of natural killer cells engineered with a HER2-targeted chimeric antigen receptor in patients with recurrent glioblastoma. Neuro. Oncol..

[49] Mantovani A., Allavena P., Sica A., Balkwill F. (2008). Cancer-related inflammation. Nature.

[50] Morrissey M.A., Williamson A.P., Steinbach A.M., Roberts E.W., Kern N., Headley M.B., Vale R.D. (2018). Chimeric antigen receptors that trigger phagocytosis. Elife.

[51] Duan Z., Li Z., Wang Z., Chen C., Luo Y. (2023). Chimeric antigen receptor macrophages activated through TLR4 or IFN-γ receptors suppress breast cancer growth by targeting VEGFR2. Cancer Immunol. Immunother..

[52] Lei A., Yu H., Lu S., Lu H., Ding X., Tan T., Zhang H., Zhu M., Tian L., Wang X. (2023). A second-generation M1-polarized CAR macrophage with antitumor efficacy. Nat. Immunol..

[53] Hrecka K., Hao C., Gierszewska M., Swanson S.K., Kesik-Brodacka M., Srivastava S., Florens L., Washburn M.P., Skowronski J. (2011). Vpx relieves inhibition of HIV-1 infection of macrophages mediated by the SAMHD1 protein. Nature.

[54] Bobadilla S., Sunseri N., Landau N.R. (2013). Efficient transduction of myeloid cells by an HIV-1-derived lentiviral vector that packages the Vpx accessory protein. Gene Ther..

[55] Gao Y., Fang X., Zhang L., Yin X. (2024). Protocol for generating human CAR-engineered macrophages by Vpx-containing lentivirus. STAR Protoc..

[56] Gao Y., Ju Y., Ren X., Zhang L., Yin X. (2023). Enhanced infection efficiency and cytotoxicity mediated by vpx-containing lentivirus in chimeric antigen receptor macrophage (CAR-M). Heliyon.

[57] Nilsson M., Ljungberg J., Richter J., Kiefer T., Magnusson M., Lieber A., Widegren B., Karlsson S., Fan X. (2004). Development of an adenoviral vector system with adenovirus serotype 35 tropism; efficient transient gene transfer into primary malignant hematopoietic cells. J. Gene Med..

[58] Ziane-Chaouche L., Raffo-Romero A., Hajjaji N., Kobeissy F., Pinheiro D., Aboulouard S., Cozzani A., Mitra S., Fournier I., Cizkova D. (2024). Inhibition of furin in CAR macrophages directs them toward a proinflammatory phenotype and enhances their antitumor activities. Cell Death Dis..

[59] Ye Z., Chen J., Zhao X., Li Y., Harmon J., Huang C., Chen J., Xu Q. (2022). In Vitro Engineering Chimeric Antigen Receptor Macrophages and T Cells by Lipid Nanoparticle-Mediated mRNA Delivery. ACS Biomater. Sci. Eng..

[60] Ohtani Y., Ross K., Dandekar A., Gabbasov R., Klichinsky M., Okamoto S., Amaishi Y., Shigeta M., Okubo Y., Ohashi Y. (2020). 128 Development of an M1-polarized, non-viral chimeric antigen receptor macrophage (CAR-M) platform for cancer immunotherapy. J. Immunother. Cancer.

[61] Chen C., Jing W., Chen Y., Wang G., Abdalla M., Gao L., Han M., Shi C., Li A., Sun P. (2022). Intracavity generation of glioma stem cell–specific CAR macrophages primes locoregional immunity for postoperative glioblastoma therapy. Sci. Transl. Med..

[62] Zhou L., Song Q., Zhang X., Cao M., Xue D., Sun Y., Mao M., Li X., Zhang Z., Liu J., Shi J. (2025). In vivo generation of CAR macrophages via the enucleated mesenchymal stem cell delivery system for glioblastoma therapy. Proc. Natl. Acad. Sci. USA.

[63] Kang M., Lee S.H., Kwon M., Byun J., Kim D., Kim C., Koo S., Kwon S.P., Moon S., Jung M. (2021). Nanocomplex-Mediated In Vivo Programming to Chimeric Antigen Receptor-M1 Macrophages for Cancer Therapy. Adv. Mater..

[64] Tang C., Jing W., Han K., Yang Z., Zhang S., Liu M., Zhang J., Zhao X., Liu Y., Shi C. (2024). mRNA-Laden Lipid-Nanoparticle-Enabled in Situ CAR-Macrophage Engineering for the Eradication of Multidrug-Resistant Bacteria in a Sepsis Mouse Model. ACS Nano.

[65] Zhang W., Liu L., Su H., Liu Q., Shen J., Dai H., Zheng W., Lu Y., Zhang W., Bei Y., Shen P. (2019). Chimeric antigen receptor macrophage therapy for breast tumours mediated by targeting the tumour extracellular matrix. Br. J. Cancer.

[66] Liu M., Liu J., Liang Z., Dai K., Gan J., Wang Q., Xu Y., Chen Y.H., Wan X. (2022). CAR-Macrophages and CAR-T Cells Synergistically Kill Tumor Cells In Vitro. Cells.

[67] Zhang L., Tian L., Dai X., Yu H., Wang J., Lei A., Zhu M., Xu J., Zhao W., Zhu Y. (2020). Pluripotent stem cell-derived CAR-macrophage cells with antigen-dependent anti-cancer cell functions. J. Hematol. Oncol..

[68] Zhang J., Webster S., Duffin B., Bernstein M.N., Steill J., Swanson S., Forsberg M.H., Bolin J., Brown M.E., Majumder A. (2023). Generation of anti-GD2 CAR macrophages from human pluripotent stem cells for cancer immunotherapies. Stem Cell Rep..

[69] Abdin S.M., Paasch D., Kloos A., Oliveira M.C., Jang M.S., Ackermann M., Stamopoulou A., Mroch P.J., Falk C.S., von Kaisenberg C.S. (2023). Scalable generation of functional human iPSC-derived CAR-macrophages that efficiently eradicate CD19-positive leukemia. J. Immunother. Cancer.

[70] Shen J., Lyu S., Xu Y., Zhang S., Li L., Li J., Mou J., Xie L., Tang K., Wen W. (2024). Activating innate immune responses repolarizes hPSC-derived CAR macrophages to improve anti-tumor activity. Cell Stem Cell.

[71] Shah Z., Tian L., Li Z., Jin L., Zhang J., Li Z., Barr T., Tang H., Feng M., Caligiuri M.A., Yu J. (2024). Human anti-PSCA CAR macrophages possess potent antitumor activity against pancreatic cancer. Cell Stem Cell.

[72] Gabitova L., Menchel B., Beghi S., Ishikawa L., Qureshi R., Best A., DeLong S., Abramson S., Condamine T., Blumenthal D., Klichinsky M. (2022). 318 Pre-clinical development of a CAR monocyte platform for cancer immunotherapy. J. Immunother. Cancer.

[73] Li N., Geng S., Dong Z.Z., Jin Y., Ying H., Li H.W., Shi L. (2024). A new era of cancer immunotherapy: combining revolutionary technologies for enhanced CAR-M therapy. Mol. Cancer.

[74] Feng M., Jiang W., Kim B.Y.S., Zhang C.C., Fu Y.X., Weissman I.L. (2019). Phagocytosis checkpoints as new targets for cancer immunotherapy. Nat. Rev. Cancer.

[75] Qian H., Fu Y., Guo M., Chen Y., Zhang D., Wei Y., Jin F., Zeng Q., Wang Y., Chai C. (2022). Dual-aptamer-engineered M1 macrophage with enhanced specific targeting and checkpoint blocking for solid-tumor immunotherapy. Mol. Ther..

[76] Reiss K.A., Angelos M.G., Dees E.C., Yuan Y., Ueno N.T., Pohlmann P.R., Johnson M.L., Chao J., Shestova O., Serody J.S. (2025). CAR-macrophage therapy for HER2-overexpressing advanced solid tumors: a phase 1 trial. Nat. Med..

[77] Gapin L. (2010). INKT cell autoreactivity: What is “self” and how is it recognized?. Nat. Rev. Immunol..

[78] Fujii S.I., Shimizu K. (2017). Exploiting antitumor immunotherapeutic novel strategies by deciphering the cross talk between invariant NKT cells and dendritic cells. Front. Immunol..

[79] Ko H.-J., Lee J.-M., Kim Y.-J., Kim Y.-S., Lee K.-A., Kang C.-Y. (2009). Immunosuppressive Myeloid-Derived Suppressor Cells Can Be Converted into Immunogenic APCs with the Help of Activated NKT Cells: An Alternative Cell-Based Antitumor Vaccine. J. Immunol..

[80] Kim C.H., Butcher E.C., Johnston B. (2002). Distinct subsets of human Vα24-invariant NKT cells: Cytokine responses and chemokine receptor expression. Trends Immunol..

[81] Maas-Bauer K., Lohmeyer J.K., Hirai T., Ramos T.L., Fazal F.M., Litzenburger U.M., Yost K.E., Ribado J.V., Kambham N., Wenokur A.S. (2021). Invariant natural killer T-cell subsets have diverse graft-versus-host-disease–preventing and antitumor effects. Blood.

[82] Simon B., Wiesinger M., März J., Wistuba-Hamprecht K., Weide B., Schuler-Thurner B., Schuler G., Dörrie J., Uslu U. (2018). The Generation of CAR-Transfected Natural Killer T Cells for the Immunotherapy of Melanoma. Int. J. Mol. Sci..

[83] Heczey A., Liu D., Tian G., Courtney A.N., Wei J., Marinova E., Gao X., Guo L., Yvon E., Hicks J. (2014). Invariant NKT cells with chimeric antigen receptor provide a novel platform for safe and effective cancer immunotherapy. Blood.

[84] Exley M.A., Friedlander P., Alatrakchi N., Vriend L., Yue S., Sasada T., Zeng W., Mizukami Y., Clark J., Nemer D. (2017). Adoptive transfer of invariant NKT cells as immunotherapy for advanced melanoma: A phase I clinical trial. Clin. Cancer Res..

[85] Li Y.R., Zhou Y., Yu J., Kim Y.J., Li M., Lee D., Zhou K., Chen Y., Zhu Y., Wang Y.C. (2025). Generation of allogeneic CAR-NKT cells from hematopoietic stem and progenitor cells using a clinically guided culture method. Nat. Biotechnol..

[86] Xu X., Huang W., Heczey A., Liu D., Guo L., Wood M., Jin J., Courtney A.N., Liu B., Di Pierro E.J. (2019). NKT cells coexpressing a GD2-specific chimeric antigen receptor and IL15 show enhanced *in vivo* persistence and antitumor activity against neuroblastoma. Clin. Cancer Res..

[87] Ngai H., Tian G., Courtney A.N., Ravari S.B., Guo L., Liu B., Jin J., Shen E.T., Di Pierro E.J., Metelitsa L.S. (2018). IL-21 Selectively Protects CD62L+ NKT Cells and Enhances Their Effector Functions for Adoptive Immunotherapy. J. Immunol..

[88] Spanoudakis E., Hu M., Naresh K., Terpos E., Melo V., Reid A., Kotsianidis I., Abdalla S., Rahemtulla A., Karadimitris A. (2009). Regulation of multiple myeloma survival and progression by CD1d. Blood.

[89] Heczey A., Xu X., Courtney A.N., Tian G., Barragan G.A., Guo L., Amador C.M., Ghatwai N., Rathi P., Wood M.S. (2023). Anti-GD2 CAR-NKT cells in relapsed or refractory neuroblastoma: updated phase 1 trial interim results. Nat. Med..

[90] Tian G., Courtney A.N., Yu H., Bhar S., Xu X., Barragán G.A., Martinez Amador C., Ghatwai N., Wood M.S., Schady D. (2025). Hyperleukocytosis in a neuroblastoma patient after treatment with natural killer T cells expressing a GD2-specific chimeric antigen receptor and IL-15. J. Immunother. Cancer.

[91] Papotto P.H., Reinhardt A., Prinz I., Silva-Santos B. (2018). Innately versatile: γδ17 T cells in inflammatory and autoimmune diseases. J. Autoimmun..

[92] Nausch N., Cerwenka A. (2008). NKG2D ligands in tumor immunity. Oncogene.

[93] Groh V., Rhinehart R., Secrist H., Bauer S., Grabstein K.H., Spies T. (1999). Broad tumor-associated expression and recognition by tumor-derived γδ T cells of MICA and MICB. Proc. Natl. Acad. Sci. USA.

[94] Dwivedi A., Fu L., Chien C.D., Pouzolles M., Shah N.N., Taylor N. (2023). Engineering Off-the-Shelf Gamma Delta CAR T Cells for the Treatment of Acute Myeloid Leukemia. Blood.

[95] Silva-Santos B., Serre K., Norell H. (2015). γδ T cells in cancer. Nat. Rev. Immunol..

[96] Makkouk A., Yang X.C., Barca T., Lucas A., Turkoz M., Wong J.T.S., Nishimoto K.P., Brodey M.M., Tabrizizad M., Gundurao S.R.Y. (2021). Off-the-shelf Vδ 1 gamma delta T cells engineered with glypican-3 (GPC-3)-specific chimeric antigen receptor (CAR) and soluble IL-15 display robust antitumor efficacy against hepatocellular carcinoma. J. Immunother. Cancer.

[97] Wculek S.K., Cueto F.J., Mujal A.M., Melero I., Krummel M.F., Sancho D. (2019). Dendritic cells in cancer immunology and immunotherapy. Nat. Rev. Immunol..

[98] Suh H.C., Pohl K., Javier A.P.L., Slamon D.J., Chute J.P. (2017). Effect of dendritic cells (DC) transduced with chimeric antigen receptor (CAR) on CAR T-cell cytotoxicity. J. Clin. Oncol..

[99] Paula-Do, Luana-Correia, Samuel-Campanelli, Ana-Carolina, Vanderson-Rocha, Théo-Gremen, Rodrigo-Nalio (2024). Generation of Dendritic Cells Expressing CAR From Induced Pluripotent Cells : An Alternative Advanced Cell Therapy For Cancer?. Hematol. Transfus. Cell Ther.

[100] Duan R., Milton P., Sittplangkoon C., Liu X., Sui Z., Boyce B.F., Yao Z. (2024). Chimeric antigen receptor dendritic cells targeted delivery of a single tumoricidal factor for cancer immunotherapy. Cancer Immunol. Immunother..

[101] Suh H.C., Pohl K.A., Termini C., Kan J., Timmerman J.M., Slamon D.J., Chute J.P. (2018). Bioengineered Autologous Dendritic Cells Enhance CAR T Cell Cytotoxicity By Providing Cytokine Stimulation and Intratumoral Dendritic Cells. Blood.

[102] Jaillon S., Ponzetta A., Di Mitri D., Santoni A., Bonecchi R., Mantovani A. (2020). Neutrophil diversity and plasticity in tumour progression and therapy. Nat. Rev. Cancer.

[103] Chang Y., Cai X., Syahirah R., Yao Y., Xu Y., Jin G., Bhute V.J., Torregrosa-Allen S., Elzey B.D., Won Y.Y. (2023). CAR-neutrophil mediated delivery of tumor-microenvironment responsive nanodrugs for glioblastoma chemo-immunotherapy. Nat. Commun..

[104] Harris J.D., Chang Y., Syahirah R., Lian X.L., Deng Q., Bao X. (2023). Engineered anti-prostate cancer CAR-neutrophils from human pluripotent stem cells. J. Immunol. Regen. Med..

[105] Majumder A., Kabir M.E., Jung H.S., Jun Y., Zhang J., Huttenlocher A., Capitini C., Thomson J., Slukvin I. (2024). iPSC-Derived CAR Neutrophils Possess Potent Activity Against Solid Tumors In Vivo. Blood.

[106] Fridman W.H., Galon J., Pagès F., Tartour E., Sautès-Fridman C., Kroemer G. (2011). Prognostic and predictive impact of intra- and peritumoral immune infiltrates. Cancer Res..

[107] Wang Y.J., Fletcher R., Yu J., Zhang L. (2018). Immunogenic effects of chemotherapy-induced tumor cell death. Genes Dis..

[108] Klapdor R., Wang S., Hacker U., Büning H., Morgan M., Dörk T., Hillemanns P., Schambach A. (2017). Improved Killing of Ovarian Cancer Stem Cells by Combining a Novel Chimeric Antigen Receptor–Based Immunotherapy and Chemotherapy. Hum. Gene Ther..

[109] Zheng H., Yang X., Huang N., Yuan S., Li J., Liu X., Jiang Q., Wu S., Ju Y., Kleeff J. (2024). Chimeric antigen receptor macrophages targeting c-MET(CAR-M-c-MET) inhibit pancreatic cancer progression and improve cytotoxic chemotherapeutic efficacy. Mol. Cancer.

[110] Obeid M., Tesniere A., Ghiringhelli F., Fimia G.M., Apetoh L., Perfettini J.L., Castedo M., Mignot G., Panaretakis T., Casares N. (2007). Calreticulin exposure dictates the immunogenicity of cancer cell death. Nat. Med..

[111] Reits E.A., Hodge J.W., Herberts C.A., Groothuis T.A., Chakraborty M., Wansley E.K., Camphausen K., Luiten R.M., De Ru A.H., Neijssen J. (2006). Radiation modulates the peptide repertoire, enhances MHC class I expression, and induces successful antitumor immunotherapy. J. Exp. Med..

[112] Luo W., Gardenswartz A., Hoang H., Chu Y., Tian M., Liao Y., Ayello J., Rosenblum J.M., Mo X., Marcondes A.M. (2024). Combinatorial immunotherapy of anti-MCAM CAR-modified expanded natural killer cells and NKTR-255 against neuroblastoma. Mol. Ther. Oncol..

[113] Chu Y., Nayyar G., Tian M., Lee D.A., Ozkaynak M.F., Ayala-Cuesta J., Klose K., Foley K., Mendelowitz A.S., Luo W. (2024). Efficiently targeting neuroblastoma with the combination of anti-ROR1 CAR NK cells and N-803 *in vitro* and *in vivo* in NB xenografts. Mol. Ther. Oncol..

[114] Ni J., Miller M., Stojanovic A., Garbi N., Cerwenka A. (2012). Sustained effector function of IL-12/15/18-preactivated NK cells against established tumors. J. Exp. Med..

[115] Gang M., Marin N.D., Wong P., Neal C.C., Marsala L., Foster M., Schappe T., Meng W., Tran J., Schaettler M. (2020). CAR-modified memory-like NK cells exhibit potent responses to NK-resistant lymphomas. Blood.

[116] Huo Y., Zhang H., Sa L., Zheng W., He Y., Lyu H., Sun M., Zhang L., Shan L., Yang A., Wang T. (2023). M1 polarization enhances the antitumor activity of chimeric antigen receptor macrophages in solid tumors. J. Transl. Med..

[117] Hosseinalizadeh H., Wang L.-S., Mirzaei H., Amoozgar Z., Tian L., Yu J. (2025). Emerging combined CAR-NK cell therapies in cancer treatment: Finding a dancing partner. Mol. Ther..

[118] Pierini S., Gabbasov R., Oliveira-Nunes M.C., Qureshi R., Worth A., Huang S., Nagar K., Griffin C., Lian L., Yashiro-Ohtani Y. (2025). Chimeric antigen receptor macrophages (CAR-M) sensitize HER2+ solid tumors to PD1 blockade in pre-clinical models. Nat. Commun..

[119] Gordon S.R., Maute R.L., Dulken B.W., Hutter G., George B.M., McCracken M.N., Gupta R., Tsai J.M., Sinha R., Corey D. (2017). PD-1 expression by tumour-associated macrophages inhibits phagocytosis and tumour immunity. Nature.

[120] Klaihmon P., Luanpitpong S., Kang X., Issaragrisil S. (2023). Anti-TIM3 chimeric antigen receptor-natural killer cells from engineered induced pluripotent stem cells effectively target acute myeloid leukemia cells. Cancer Cell Int..

[121] Korde N., Carlsten M., Lee M.J., Minter A., Tan E., Kwok M., Manasanch E., Bhutani M., Tageja N., Roschewski M. (2014). A phase II trial of pan-KIR2D blockade with IPH2101 in smoldering multiple myeloma. Haematologica.

[122] Zhang H., Huo Y., Zheng W., Li P., Li H., Zhang L., Sa L., He Y., Zhao Z., Shi C. (2024). Silencing of SIRPα enhances the antitumor efficacy of CAR-M in solid tumors. Cell. Mol. Immunol..

[123] Chen S., Wang Y., Dang J., Song N., Chen X., Wang J., Huang G.N., Brown C.E., Yu J., Weissman I.L. (2025). CAR macrophages with built-In CD47 blocker combat tumor antigen heterogeneity and activate T cells via cross-presentation. Nat. Commun..

[124] Ma R., Lu T., Li Z., Teng K.Y., Mansour A.G., Yu M., Tian L., Xu B., Ma S., Zhang J. (2021). An oncolytic virus expressing il15/il15ra combined with off-the-shelf egfr-car nk cells targets glioblastoma. Cancer Res..

[125] Osali A., Zhiani M., Ghaebi M., Meymanat M., Esmaeilzadeh A. (2020). Multidirectional Strategies for Targeted Delivery of Oncolytic Viruses by Tumor Infiltrating Immune Cells. Pharmacol. Res..

[126] He J., Munir F., Ragoonanan D., Zaky W., Khazal S.J., Tewari P., Fueyo J., Gomez-Manzano C., Jiang H. (2023). Combining CAR T Cell Therapy and Oncolytic Virotherapy for Pediatric Solid Tumors: A Promising Option. Immuno.

[127] Labani-Motlagh A., Naseri S., Wenthe J., Eriksson E., Loskog A. (2021). Systemic immunity upon local oncolytic virotherapy armed with immunostimulatory genes may be supported by tumor-derived exosomes. Mol. Ther. Oncolytics.

[128] Sharp D.W., Lattime E.C. (2016). Recombinant Poxvirus and the tumor microenvironment: Oncolysis, immune regulation and immunization. Biomedicines.

[129] Li G., Wu X., Chan I.H., Trager J.B. (2020). Abstract 4235: A combination of CAR-NK and CAR-T cells results in rapid and persistent anti-tumor efficacy while reducing CAR-T cell mediated cytokine release and T-cell proliferation. Cancer Res..

[130] Netea M.G., Domínguez-Andrés J., Barreiro L.B., Chavakis T., Divangahi M., Fuchs E., Joosten L.A.B., van der Meer J.W.M., Mhlanga M.M., Mulder W.J.M. (2020). Defining trained immunity and its role in health and disease. Nat. Rev. Immunol..

[131] Baldwin J.G., Heuser-Loy C., Saha T., Schelker R.C., Slavkovic-Lukic D., Strieder N., Hernandez-Lopez I., Rana N., Barden M., Mastrogiovanni F. (2024). Intercellular nanotube-mediated mitochondrial transfer enhances T cell metabolic fitness and antitumor efficacy. Cell.

